# DNLA Delayed the Appearance of Learning and Memory Impairment of APP/PS1 Mice: Involvement of mTOR/TFEB/v‐ATPase Signaling Pathway

**DOI:** 10.1111/cns.70300

**Published:** 2025-03-06

**Authors:** Yajuan Wu, Xuejia Liu, Guohui Luo, Qiye Li, Bin Guo, Lisheng Li, Jing Nie

**Affiliations:** ^1^ Key Laboratory of Basic Pharmacology of Ministry of Education Zunyi Medical University Zunyi China

**Keywords:** alkaloids from *Dendrobium nobile* Lindl, Alzheimer's disease, autophagic lysosomes, lysosomal biogenesis, v‐ATPase

## Abstract

**Introduction:**

Alzheimer's disease (AD) is a progressive neurodegenerative disorder with cognitive impairment that currently is incurable. There is existing evidence to suggest that vacuolar adenosine triphosphatase (v‐ATPase) is one of the early key driving factors in the pathological process of AD. Thus, early intervention of v‐ATPase may be a viable strategy.

**Aims:**

Observing whether early intervention with DNLA can delay learning and memory impairment in mice, and further exploring the mechanism of DNLA delaying AD in vitro based on v‐ATPase.

**Methods:**

Four‐month‐old APP/PS1 transgenic mice were treated with alkaloids from Dendrobium nobile Lindl (DNLA) 20 and 40 mg/kg/day for 5 months. The Morris water maze test and nest test showed that DNLA administration significantly delayed the appearance of cognitive deficits in APP/PS1 mice. We further investigated the mechanism of DNLA promoting lysosome acidification in vitro by using PC12 cells.

**Results:**

We found that DNLA increases the degradation of β‐amyloid (Aβ) contained in the autophagic lysosomes and alleviates the aging of neurons by promoting lysosome acidification and improving autophagy flow. In PC12 cells, DDB could promote the separation of mTOR and lysosome, promote the nuclear translocation of transcription factor EB (TFEB), and then promote lysosome biogenesis and lysosome acidification by targeting ATP6V1A.

**Conclusion:**

These results unraveled that preventive administration of DNLA may delay the impairment of learning and memory in APP/PS1 mice. The molecular mechanism may be related to promoting the mTOR‐TFEB‐v‐ATPase pathway.

## Graphical abstract

The article unraveled that preventive administration of DNLA may delay the impairment of learning and memory in APP/PS1 mice. The molecular mechanism may be related to promoting the mTOR‐TFEB‐v‐ATPase pathway.

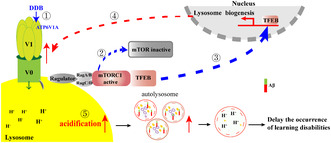

## 1 Introduction

Alzheimer's disease (AD) is a central nervous system degenerative disease characterized by progressive cognitive impairment and memory impairment, the most typical pathological features of which are extracellular amyloid plaques and intracellular neurofibrillary tangles [1]. At present, a series of drugs targeting these two toxic aggregates have been developed, but unfortunately, none have significantly improved the primary clinical endpoint of cognitive function, and some were terminated due to serious side effects [2, 3, 4]. It has been reported that the development of AD is a continuous pathological process, and the pathological changes of AD patients have already appeared in the first 15–20 years of their clinical symptoms [5]. Therefore, early identification of pathological changes and therapeutic intervention in the asymptomatic period may be an effective strategy for the prevention and treatment of AD.

Abnormalities in the endosome‐lysosomal system are one of the earliest detectable histopathological features of AD [6]. Ju‐hyun Lee demonstrated that in five different AD models, autophagy failure resulted from a decrease in the acidification of early lysosomes and autolysosomes [7, 8]. While the maintenance of the acidic environment in the lysosomal cavity mainly depends on vacuolar adenosine triphosphatase (v‐ATPase) [9], the v‐ATPase uses the energy from ATP hydrolysis to pump H^+^ into the lysosome and maintains the pH values in the lumen in the appropriate range [10]. Therefore, abnormal v‐ATPase function will lead to impaired lysosomal acidification. As a result, abnormal v‐ATPase function may be one of the key factors in the early pathogenesis of AD, and importantly, early intervention via v‐ATPase may delay AD.

Alkaloids from *Dendrobium nobile* Lindl (DNLA) are one of the main active ingredients extracted from *Dendrobium nobile* Lindl (DNL). Dendrobine (DDB) (98.0%) is the main component of DNLA. Our previous studies have indicated that DNLA can improve the learning and memory impairment of APP/PS1 mice [11] and AD model animals caused by the injection of lipopolysaccharide [12] and Aβ_25–35_ [13]. In addition, our previous study found that DNLA could increase the protein level of the ATP6V1A and promote lysosomal acidification [11]. Therefore, we sought to observe the changes in v‐ATPase in early APP/PS1 mice and also to observe the effect of DNLA intervention on the learning and memory dysfunction in APP/PS1 mice. Moreover, an additional aim was to further analyze its mechanism both upstream and downstream of v‐ATPase.

## 2 Materials and Methods

### 2.1 Reagents and Antibodies

DNL was purchased from Xintian Traditional Chinese Medicine Industry Development Co Ltd. (Guizhou, China). DNLA was isolated from the extracts and analyzed by LC/MS. Alkaloids accounted for 86.4% of the DNL, and DDB (C_16_H_25_O_2_N) accounted for 93.1% of DNLA [13]. DDB monomer was purchased from Chengdu Alfa Biotechnology Co. Ltd. (Chengdu, China). Thioflavine‐S dye (#T1892) was purchased from Sigma. Aβ_1–40_ (#12990T) and Aβ_1–42_ (#14974T) were purchased from Cell Signaling; LysoTracker Red (#L8010) was purchased from Beijing Solarbio Technology Co. Ltd. (Beijing, China). Primary antibodies against p62 (#18420‐1‐AP), LC3 (#14600‐1‐AP), TFEB (#13372‐1‐AP), Lamp2(#2D3B9) and GAPDH (#60004‐1‐Ig) and β‐actin (#60008‐1‐lg) were purchased from Proteintech Group (Wuhan, China). FKBP12 (#55104) was obtained from Cell Signaling Technologies (Danvers, MA, USA). In addition, mTOR (#A2445) was purchased from Abclonal Technology (Wuhan, China). ATP6V1A (#GTX633542) and ATP6V0a1 (#GTX44653) were purchased from GeneTex (USA). Finally, p‐mTOR (#AF3308), p70S6K (#AF6226), p‐p70S6K (#AF3228), and PNCA (#AF0239) were obtained from Affinity (USA).

### 2.2 Mouse Lines and Cell Care

Male APPswe/PS1E9 transgenic (APP/PS1) mice and their wild‐type (WT) littermates [Certificate no: SCXK (Su) 2016‐0010] were purchased from the Cavance Laboratory Co. Ltd of Changzhou province (China). The mice were housed in SPF grade animal facilities [Certificate No. SYXK (Qian) 2021‐0003] at 22°C ± 3°C with a 12 h light/dark cycle. Mice were provided free access to food and water. All animal procedures were approved by the Animal Experimentation Ethics Committee of Zunyi Medical University (Zunyi, China).

The rat pheochromocytoma PC12 cell lines used in this study were purchased from Shanghai Gaining Biotechnology Co. Ltd. (China). The cell line was cultured in DMEM (10% FBS) medium supplemented with 10% fetal bovine serum and 2% double antibodies. Cells were routinely cultured at 37°C in a 5% CO_2_ incubator. During serum starvation experiments, cells were cultured in EBSS medium.

### 2.3 Experimental Design

Project 1: Male 4‐month‐old APP/PS1 mice and WT mice were divided into the APP/PS1 group and the WT group.

Project 2: Male 4‐month‐old APP/PS1 mice were randomly divided into three groups: the DNLA groups (20 and 40 mg/kg), the APP/PS1 group (*n* = 10); age‐matched WT male mice comprising the WT control group and the DNLA group (*n* = 10). Once daily for 5 months, mice in the administration group were given DNLA 20 mg/kg/day and 40 mg/kg/day; the WT group and the APP/PS1 group were given an equal volume of vehicle (0.1% (v/v) Tween‐80 double distilled water).

Project 3: PC12 cells were randomly divided into control and different concentrations of DDB administration groups.

Project 4: Construction of PC12 cells that silence ATP6V1A (Sh‐ATP6V1A), and the cells were randomly divided into the Sh‐Control group, Sh‐Control+DDB group, Sh‐ATP6V1A group, and Sh‐ATP6V1A + DDB group.

### 2.4 Morris Water Maze Experiments

The spatial learning and memory ability of APP/PS1 mice was measured by the Morris water maze (MWM). The MWM experiment was conducted in a 120 cm white pool with a 10 cm escape platform placed 1 cm below the water surface in the center of the target quadrant, and data were recorded and analyzed by the TopView Animal Behavior Analyzing System (Version 3.00). The MWM is divided into two steps, with the place navigation test being the first step. Mice were released from one of three quadrants without platforms. Each trial lasted for 60 s or ended as soon as the mouse climbed onto the platform. The time required for the mice to climb onto the platform within 60 s was recorded as the escape latency. We regarded the time required as 60 s if the mouse failed to find the platform within 60 s. The spatial probe test was the second step and was performed on day 5. The platform was removed, and each mouse was allowed to swim for 60 s in the pool. Simultaneously, the searching distance, searching time, swimming speed, and frequency crossing the target quadrant were measured [11].

### 2.5 Nest Building

The mice were divided into single cages (one animal in each cage). After 48 h of adaptation to the environment, each cage was replaced with wood shavings with a thickness of 1 cm, and 10 square unscented paper towels of the same size were evenly spread. After 24 h, the nesting ability of the mice was scored and photographed by three people independently in a double‐blind situation according to the improved 6‐point method. The scoring rule was to divide the whole rectangular squirrel cage into six areas and then add up all of the areas covered by the paper to calculate the total score.

### 2.6 Thioflavine‐S Staining

Slides were rewarmed at 37°C for 20 min and covered with 100 μL of 1% Thioflavine‐S, shielded from light for 20 min, and rinsed with PBS and eluted with 75% (v/v) ethanol. Senile plaques were observed with a 4× positive fluorescence microscope. Three brain slices of the same coronal section were taken, and Image J software was used to analyze the senile plaques in the hippocampus.

### 2.7 Hematoxylin and Eosin Staining

After the MWM test, three mice were randomly selected from each group and anesthetized with 2% sodium pentobarbital and then perfused with phosphate‐buffered saline (PBS; 0.1 M, 4°C) via the ascending aorta, followed by 4% paraformaldehyde until the tail and limbs were rigid. Thereafter, the brain tissues were removed and bisected in half. Then, half of the brain tissue was fixed and embedded with paraffin wax, sliced (4 μm) with a microtome, and then stained with an HE dyeing machine. Thereafter, the histological change was observed under the microscope (BX 43 Olympus, Tokyo, Japan).

### 2.8 β‐Galactosidase (SA‐β‐Gal) Staining

The brain slices are covered with 20 μL of dye fixing solution and fixed for 15 min. Wash tissue with PBS for three times. 30 μL of dyeing solution (A: 10 μL, B: 10 μL, C: 930 μL, X‐gal:50 μL) was added, incubated at 37°C overnight, and observed under a 4× ordinary optical microscope (BX 43 Olympus, Tokyo, Japan).

### 2.9 Immunofluorescence Staining

The brain slices (30 μm thick) and cells were washed 3 times with PBS, fixed with 4% paraformaldehyde for 20 min, dipped in 0.3% (w/v) Triton X‐100 for 15 min, and then blocked in goat serum for 30 min. After washing again with PBS, the slices were treated with the appropriate primary antibodies diluted in the blocking solution at 4°C overnight. The antibodies used were as follows: rabbit anti‐LC3 (1:200), anti‐Aβ_1–42_ (1:200), anti‐v‐ATPase (1:200), and anti‐M6PR (1:200); rat anti‐LAMP2 (1:200); and mouse anti‐cat D (1:200). The slices were incubated with donkey anti‐rabbit Alexa 488 (1:1000), goat anti‐rat IgG (H + L) (1:1000), and goat anti‐mouse IgG (H + L) (1:1000) for 1 h at 37°C. After washing with PBS, the slices were covered with glass slides. Images were acquired using a positive fluorescence microscope (Olympus, Tokyo, Japan).

### 2.10 Western Blot (WB) Analysis

Protein samples were extracted from the hippocampus of the mouse using RIPA buffer supplemented with the protease inhibitor PMSF. After incubation and centrifugation (12,000 × *g*, 15 min, 4°C), the supernatant was collected, and total protein levels were quantified using BCA Assay Kits (Beyotime, P0010). Equal amounts of total protein (30 μg) were separated on a 10% sodium dodecyl sulfate polyacrylamide (SDS‐PAGE) gel, and the separated proteins were transferred to a PVDF membrane. Following that, the membrane was incubated at 4°C overnight with antibodies against primary antibodies. Subsequently, the membranes were incubated with species‐specific HRP‐conjugated secondary antibodies at room temperature for 3 h. The blots were visualized using an enhanced chemiluminescence WB detection kit (7Sea Biotech, China) and imaged using the Gel Imaging system (Bio‐Rad Laboratories Inc., Hercules, CA, USA). Band intensities were quantified by densitometry using Image Lab software.

### 2.11 Lysosomal Acidity Assay

Cells were subjected to drug intervention, and LysoTracker Red working solution was added 2 h before the end of this intervention. The medium that contained the LysoTracker Red was removed after incubation and washed three times with PBS, fixed at room temperature for 20 min with 4% paraformaldehyde, and then observed under an inverted fluorescence microscope. Three replicate wells were set up for each group, and five random fields of view were taken for each well.

### 2.12 qRT‐PCR

Total RNA was extracted from the cells using Trizol. RNA samples were then reverse transcribed using a PrimeScript TM RT Master MIX (#R036A, TaKaRa) kit according to the manufacturer's instructions. Quantitative PCR was performed using an iQTMSYBR Green Supermix (#1708880B10, Bio‐RAD) kit with the following primer sequences, all synthesized by Bio‐Engineering Corporation: LAMP1 (FW, 5′‐CAGGGTAGAAAGTGACAGGTTTGGG‐3′; RV, 5′‐AGGTAGGCGATGAGGACGATGAG‐3′), CSTB (FW, 5′‐CGCCGACAAGGTGAAGTCTCAAC‐3′; RV, 5′‐GGCCACTACCTGTCTCCTGAAGG‐3′), ATP6V1A (FW, 5′‐CCGTACTCCGCACTGGTAAAC‐3′; RV, 5′‐TGGGGATGTAGATACTTTGGGT‐3′), ATP6V0A1 (FW, 5′‐ATGGATCGAAAACTCCGATTTGT‐3′; RV, 5′‐TTCTCGAAATTGGCCTCTCTAAGTC‐3′), β‐actin (FW, 5′‐CACCCGCGAGTACAACCTTC‐3′; RV, 5′‐CCCATACCCACCATCACACC‐3′). All mRNA levels of these genes were normalized to the expression of endogenous β‐actin, and relative expression levels were calculated using the 2^(−ΔΔCT)^ method.

### 2.13 Establishment of the Lentiviral Vector ATP6V1A‐shRNA

The three top‐scoring shRNA sequences targeted for rat ATP6V1A were designed and synthesized by Hanbio Biotechnology using the pHBLV‐U6‐MCS‐CMV‐ZsGreen‐PGK‐PURO vector. In addition, a green fluorescent protein‐tagged lentiviral vector was constructed to evaluate the efficiency of transfection. Such an empty vector was used as a negative control. The initial lentiviral vector titer contained 1.5 × 10^8^ TU/mL.

### 2.14 HBAD‐mcherry‐LC3 Assay

The PC12 cells were inoculated in 24‐well plates and incubated overnight with 250 μL of fresh medium and 15 μL of HBAD‐mcherry‐LC3 adenovirus at a titer of 1 × 10^9^ PFU/mL per well (Hanbio Biotechnology, Shanghai, China). The wells were then incubated for 4 h and replenished to a 500 μL culture volume. Images were collected 24 h after infection using confocal microscopy.

### 2.15 Drug Affinity Responsive Target Stability (Darts) [14]

The lysate of mouse hippocampal tissue and PC12 cells was diluted to 2 mg/mL with TNC buffer (50 mM Tris–HCl, 50 mM NaCl, 10 mM CaCl_2_) and treated with 100 mM DDB or DMSO. After incubation for 2 h at 4°C, 1 μg/mL Pronase E (#P8360, Solarbio) was added to the samples for 20 min. After 20 min had elapsed, the reaction was immediately terminated by loading buffer, and the samples were assayed by WB.

### 2.16 Statistical Analysis

SPSS29.0 statistical analysis software was used for all experimental data. All data are presented as the mean ± standard deviation ($\bar{\chi }$ ± SD) and tested for normality. One‐way ANOVA or independent sample *T* test was used for data conforming to normal distribution, while a non‐parametric test was used for data not conforming to normal distribution. The MWM test data were analyzed by repeated‐measures analysis of variance, and the other data were compared using one‐way analysis of variance (ANOVA) followed by the LSD test or Dunnett T3 test (the variance is uneven). The comparison between the two groups was performed using the independent sample *t* test, with *p* < 0.05 suggesting statistical significance.

## 3 Results

### 3.1 Changes in v‐ATPase Occurred Before the Onset of Learning and Memory Impairment in APP/PS1 Mice at 4 Months of Age

**FIGURE 1:**
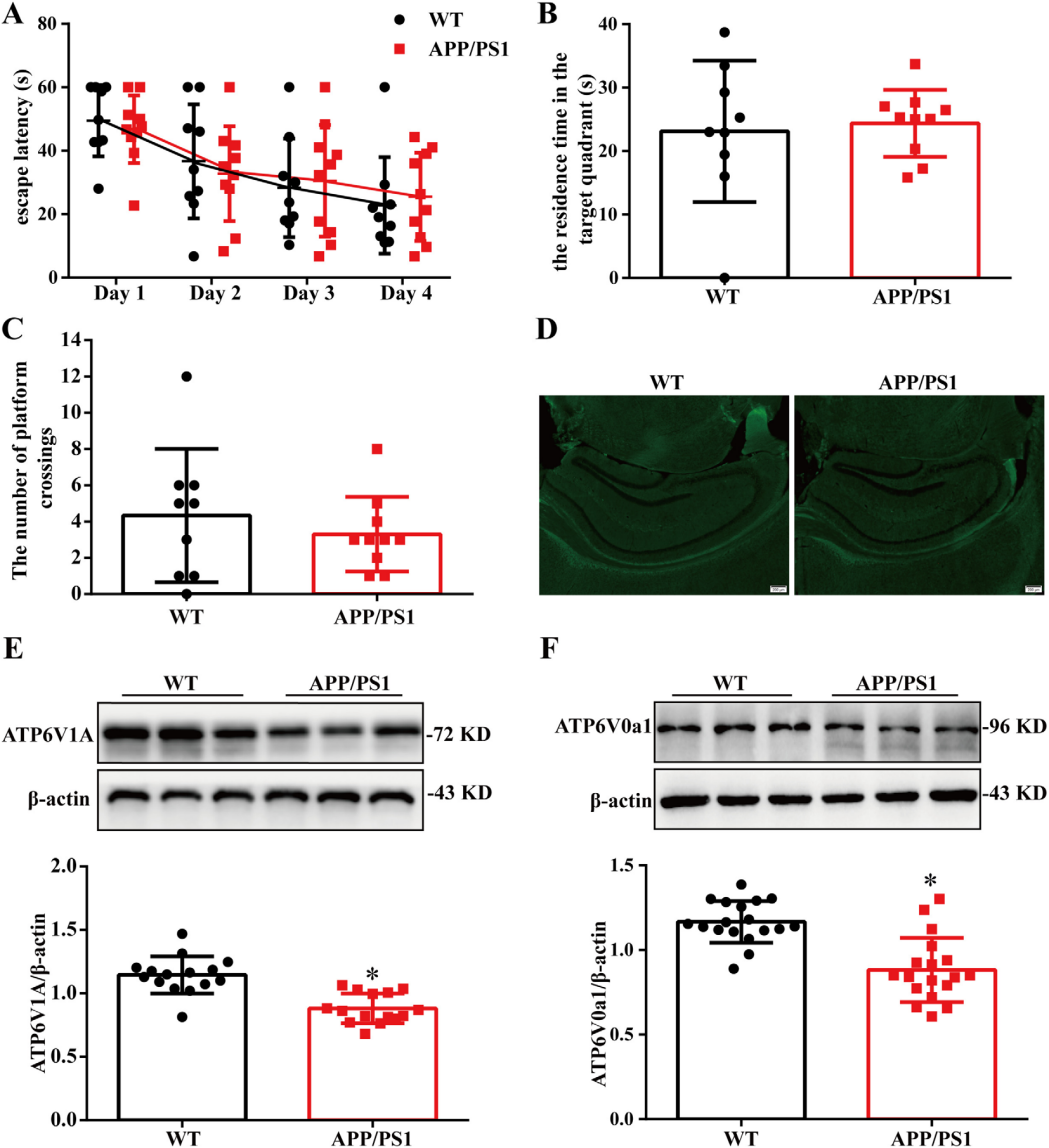
Spatial learning and memory function of 4‐month‐old mice. (A) The escape latency of mice to reach the hidden platform from day 1 to day 4. (B) The residence time in the target quadrant. (C) The number of platform crossings. ($\bar{\chi }$ ± SD, *n* = 9–10) (D) Senile plaques in the hippocampus of 4‐month‐old mice (Scale = 200 μm, *n* = 3). (E and F) Representative bands quantification of protein expression level of ATP6V0a1, ATP6V1A. ($\bar{\chi }$ ± SD, *n* = 6), *p < 0.05 vs. WT group.

The MWM test was used to detect the spatial learning and memory ability of 4‐month‐old APP/PS1 mice. We found that there were no significant differences in the target quadrant residence time and the number of platform crossings between the two groups (Figure 1A–C). Thioflavin‐S staining was used to observe the changes in amyloid plaques in the brains of APP/PS1 mice. The results showed that there was no amyloid plaque deposition in the brains of 4‐month‐old mice (Figure 1D).

The v‐ATPase is a polysubunit polymerase composed of subunits V0 and V1. Studies have reported that the v‐ATPase subunit is implicated in AD [15]. The PS1 whole protein is a special ligand of the V0a1 subunit of v‐ATPase and is required for *n*‐glycosylation, stabilization, and anchoring to lysosomes of the V0a1 subunit. A lack of PS1 leads to increased lysosomal pH, impaired lysosomal proteolysis, and pathological changes of AD‐like autophagosomes [8]. In the case of sporadic AD, researchers reported that the ATP6V1A was down‐regulated in the brain tissues of patients with sporadic AD, and its expression was negatively correlated with the clinicopathological features of AD [16]. WB was used to detect the changes in the v‐ATPase of 4‐month‐old APP/PS1 mice. The results showed that compared to WT mice, ATP6V1A and ATP6V0a1 protein expressions in the hippocampus of the APP/PS1 group were significantly decreased (Figure 1E,F).

### 3.2 DNLA Delayed the Development of Learning and Memory Impairment and Retarded the Aging of Hippocampal Neurons in APP/PS1 Mice

**FIGURE 2:**
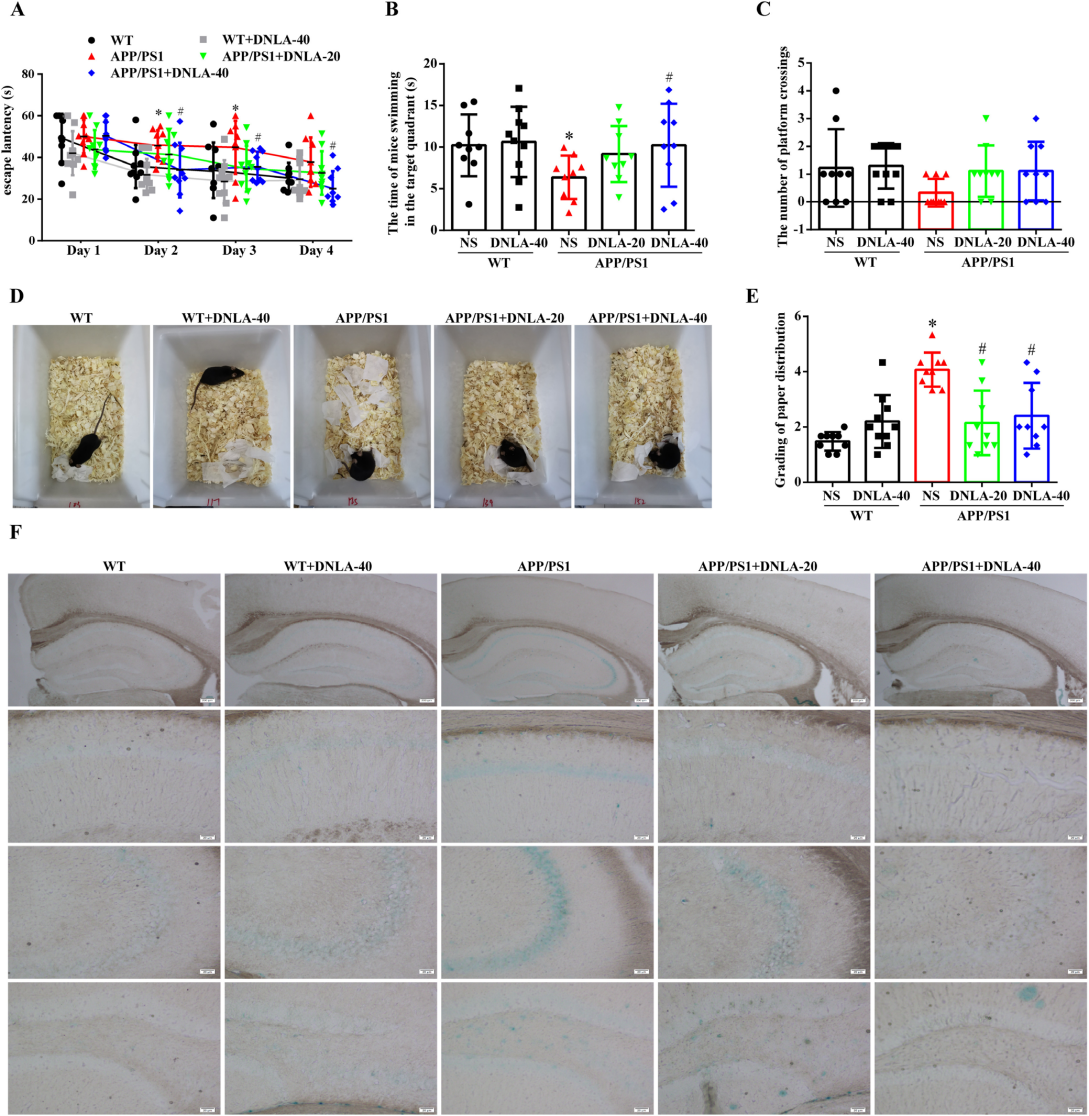
Results of learning and memory ability in 9‐month‐old mice. (A) The escape latency from day 1 to day 4. (B) The time of mice swimming in the target quadrant. (C) The number of times the mice crossed the platform. (D) Image of mice nesting behavior. (E) Scoring statistics of nesting ability in mice ($\bar{\chi }$ ± SD, *n* = 9–10), *p < 0.05 vs. WT group, ^#^ *p* < 0.05 vs. APP/PS1 group. (F) Representative images of β‐galactosidase kit staining (*n* = 3, scale = 200 μm, 20 μm).

After 5 months of intragastric administration of DNLA, the Morris water maze was used to observe the effect of DNLA on the learning and memory of 9‐month‐old APP/PS1 mice. Compared to the WT group, the average escape latency of the APP/PS1 group significantly increased, while it was significantly decreased after administration with DNLA‐40 (Figure 2A). During the space exploration period on the fifth day, the time spent in the target quadrant and the number of platform crossings of the mice in the APP/PS1 group were significantly reduced compared to the WT group and were significantly increased in the DNLA‐40 group, with no significant differences compared to the WT group (Figure 2B,C). As an indicator of early behavioral defects [17], nesting behavior is controlled by many brain regions and neurotransmitter activities, which can reflect the daily behavioral ability of mice. The result of the nest‐building experiment showed that the paper in the WT group was concentrated, while the paper in APP/PS1 mice was dispersed, indicating that the nesting ability of mice in the APP/PS1 group was reduced compared to the WT group. After DNLA administration, the nesting ability of mice in the APP/PS1 group was significantly improved, showing no statistical differences with the WT group (Figure 2D,E).

As one of the pathological characteristics of AD, the decrease in the number of neurons plays a crucial role in the cognitive function of mice [2]. To observe the effect of DNLA on the morphology of neurons, the morphology of hippocampal neurons was observed by HE and Nissl staining. The results showed that the morphology and structure of neurons in each group were complete and neatly arranged, and there were no significant differences in the morphology and number of neurons (Figure S1). Aging is a major risk factor in most cases of AD [18, 19]; β‐galactosidase (SA‐β‐gal) is the most widely used biomarker to identify senescent cells in vitro and in vivo, and its activity is highly correlated with senescent cells [20]. The activity of β‐galactosidase can be detected by cytochemical analysis, and its substrate 5‐bromo‐4‐chloro‐3‐indoyl β‐d‐galactopyranoside (X‐Gal), which is cleaved by β‐galactosidase, produces an insoluble blue compound [21]. In order to detect whether there were functional abnormalities, the number of senescent cells in the mouse brain was detected by β‐galactosidase kits. The results showed that compared with the WT group, the dark blue staining in the hippocampal CA1, CA3, and DG regions of APP/PS1 mice was significantly increased. After DNLA administration, the area of blue in APP/PS1 mice was significantly reduced (Figure 2F).

### 3.3 DNLA Decreased the Content of Intracellular Aβ in the Hippocampi of APP/PS1 Mice

**FIGURE 3:**
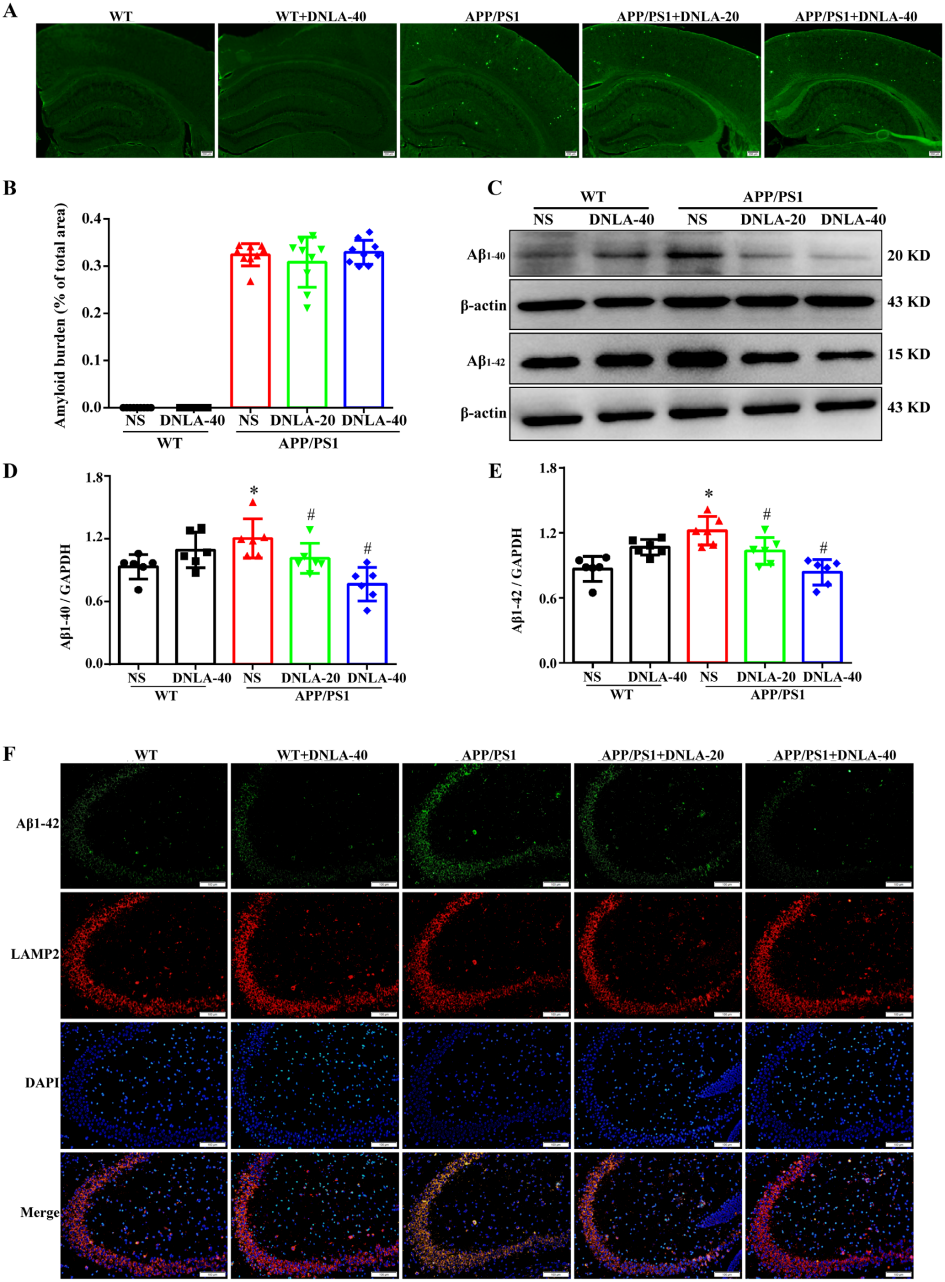
Effect of DNLA on Aβ of the hippocampus in 9‐month‐old APP/PS1 mice. (A) Representative image of thioflavin staining (scale = 200 μm, *n* = 3). (B) statistical plots of Aβ plaques content in each group. (C) Representative bands of Aβ_1–40_, Aβ_1–42_. ($\bar{\chi }$ ± SD, *n* = 6), *p < 0.05 vs. WT group, ^#^ *p* < 0.05 vs. APP/PS1 group. (D and E) Quantification of the protein expression level of Aβ_1–40_, Aβ_1–42_. (F) Effects of DDB on Aβ_1–42_ in hippocampal lysosomes of 9‐month‐old APP/PS1 mice. (Scale = 100 μm, *n* = 3).

The extracellular senile plaque deposition in APP/PS1 mice was observed by Thioflavine‐S staining. The results showed that compared with the control group, the extracellular amyloid plaque deposition in the hippocampus of APP/PS1 mice was significantly increased, and there was no significant reduction in extracellular plaque deposition after DNLA administration (Figure 3A,B). Furthermore, WB data showed that compared with the WT group, the protein levels of Aβ_1–40_ and Aβ_1–42_ in the hippocampus of the APP/PS1 group were significantly increased and were significantly lower after DNLA administration. Moreover, there was no significant differences compared with the WT group (Figure 3C–E). These results suggest that DNLA may reduce intracellular Aβ but not prevent the production of extracellular amyloid plaques. Thus, intracellular Aβ expression was further determined. Lysosomes are the major organelles that degrade Aβ [22]; therefore, immunofluorescence was used to observe the co‐localization of lysosomal marker protein Lamp2 and Aβ_1–42_. The results showed that the co‐localization of Aβ_1–42_ and lysosomal markers in the APP/PS1 group was significantly higher than that in the WT group, and it was significantly lower after DNLA administration. In addition, there were no significant differences between the APP/PS1 group and the WT group (Figure 3F). These results suggest that DDB may delay neuronal senescence by promoting the clearance of intracellular Aβ.

### 3.4 DNLA Promoted Lysosomal Acidification and Improved Autophagic Flow in the Hippocampi of APP/PS1 Mice

**FIGURE 4:**
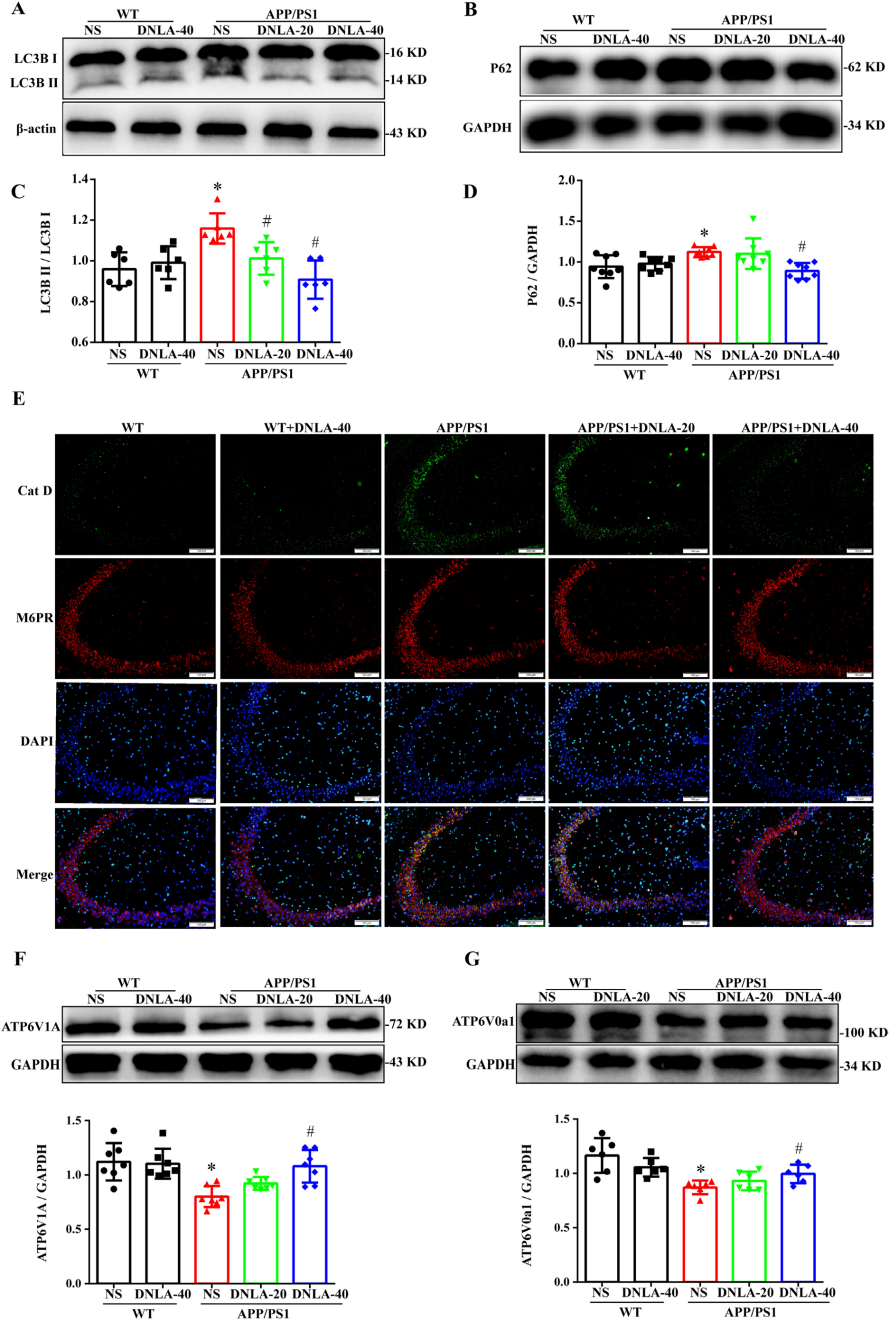
Effects of DNLA on proteins associated with the autophagy lysosome pathway. (A) Representative bands of LC3 and p62. (B and C) Quantification of protein expression levels of LC3 and p62. (D) Hippocampal lysosomal acidity in 9‐month‐old APP/PS1 mice. (E) Colocalization of cathepsin D and M6PR. (F and G) Representative bands and quantification of protein expression levels of ATP6V1A and ATP6V0a1. ($\bar{\chi }$ ± SD, *n* = 6), *p < 0.05 vs. WT group, ^#^ *p* < 0.05 vs. APP/PS1 group.

The autophagy‐lysosome pathway is the main way to degrade harmful substances such as Aβ [23]. To further explore the mechanism of DNLA's clearance of intracellular Aβ, we further determined the protein expression of the autophagy‐related proteins LC3 and P62. LC3 is generated when autophagy is activated, and P62 is degraded when the autophagosome and lysosome are fused [24, 25]. The results showed that compared to the WT group, the expression of LC3 and P62 in the APP/PS1 group mice was significantly increased. Furthermore, DNLA preventive administration was shown to reduce the protein expression of LC3 and P62, and there were no significant differences compared to the WT group (Figure 4A–D).

After the fusion of autophagosomes and lysosomes, the autophagosomes are degraded by the hydrolytic enzymes in the lysosomes. Studies have confirmed that abnormal autophagy in AD patients mainly occurs at the stage of autophagolysosomal proteolysis [26]. Cathepsin D (Cat D), the major aspartic protease of lysosomes, is synthesized in the endoplasmic reticulum and subsequently generates an inactive proteinase that binds to mannose‐6‐phosphate (M‐6‐P) on the Golgi apparatus. Cat D is recognized by the M‐6‐P receptor on the Golgi apparatus and transported into lysosomes. Under the acidic environment of lysosomes, Cat D is separated from M6PR and undergoes several proteolytic processes to finally produce mature proteolytic enzymes [6]. To observe the effect of DNLA on lysosomal hydrolase, double immunofluorescence labeling was performed with Cat D and M6PR antibodies. The results showed that, compared to the WT group, the fluorescence co‐localization of Cat D and M6PR was significantly increased in APP/PS1 mice, while the fluorescence co‐localization of Cat D and M6PR was significantly decreased after high‐dose DNLA administration. In addition, there were no significant differences between APP/PS1 mice and the WT group (Figure 4E). These data suggested that DNLA can improve autophagy flow by increasing lysosomal enzyme activity.

The v‐ATPase is the main proton pump that regulates the acidity of lysosomes, by controlling the entry of H^+^ by regulating the binding and dissociation of its V1 and V0 subunits [27, 28]. The expression of v‐ATPase subunits in the hippocampus of APP/PS1 mice was detected by WB. The results showed that compared to the WT group, the protein expression of ATP6V1A and ATPV0a1 in the APP/PS1 group mice was significantly decreased, while the protein expression of ATP6V1A and ATPV0a1 was significantly increased after DNLA administration. There were no statistically significant differences compared to the WT group (Figure 4F,G). These data also suggested that DNLA up‐regulated the expression of V0 and V1 subunits to promote lysosome acidification and improve autophagy flow.

### 3.5 DDB Promoted Lysosomal Acidification and Lysosomal Biogenesis

**FIGURE 5:**
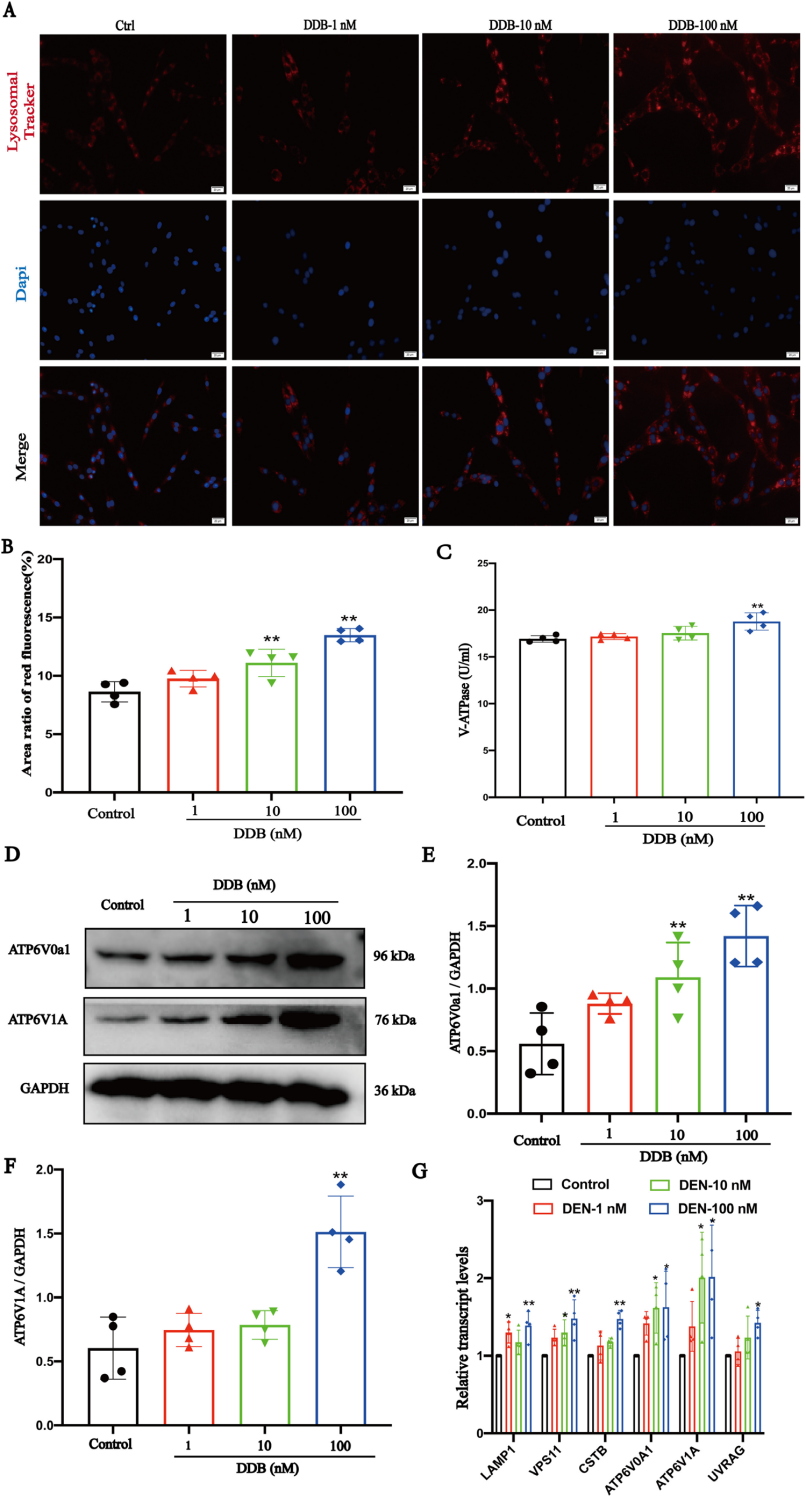
The effect of DDB on lysosomal acidification and v‐ATPase. (A and B) The effect of DDB on lysosomal acidification was detected by LysoTracker Red. (C) The effect of DDB on v‐ATPase activity in PC12 cells. (D, E and F) The effect of DDB on the protein expression of ATP6V0a1 and ATP6V1A. ($\bar{x}$ ± SD, *n* = 3); (G) The effect of DDB on the autophagy and lysosome related genes. ($\bar{x}$ ± SD, *n* = 4) *p < 0.05, **p < 0.01 vs. Control group.

DDB is the characteristic component in DNLA. In this study, the content of DDB in the DNLA we used was 93.1%. In vitro experiments, we found that both DNLA and DDB could increase the protein level of ATP6V1A, and there was no significant difference (Figure S2); DDB is very likely to be the material basis for DNLA to exert its curative effect. Therefore, we chose DDB to conduct a mechanism study, on the one hand, to clarify the anti‐AD mechanism of DNLA, and on the other hand, to clarify the material basis of DNLA's anti‐AD effect. We observed the effect of DDB on lysosome acidification by the LysoTracker Red method [29, 30]. The red fluorescence intensity of DDB dose groups was significantly higher than that of the control group in PC12 cells (Figure 5A,B), and we obtained the same result on HT22 cells (also called mouse hippocampal neuron cells, which is immortalized mouse hippocampal cell lines subcloned from the HT‐4 cell lines) (Figure S3), which suggests that DDB promotes lysosomal acidification.

We also tested the effect of DDB on v‐ATPase activity by ELISA and found that DDB (100 nM) significantly increased v‐ATPase activity compared to the control group (Figure 5C). WB was used to detect the effect of DDB on the protein levels of some v‐ATPase subunits in the PC12 cells (Figure 5D–F). The results showed that DDB significantly increased the protein levels of ATP6V1A and ATP6V0a1, and the same phenomenon was observed in HT22 cells and primary hippocampal neurons (Figure S4).

In order to further explore the mechanism of DDB up‐regulating v‐ATPase protein levels, RT‐PCR was used to examine the levels of v‐ATPase and autophagolysosomal‐related genes, including the lysosomal hydrolase auxiliary protein CSTB, the lysosomal membrane protein LAMP1, VSP11, UVRAG, and the v‐ATPase‐related subunits ATP6V0A1 and ATP6V1A (Figure 5G). Our results showed that DDB upregulated the transcript levels of these genes in PC12 cells, and we obtained the same result in HT22 cells (Figure S5), which suggests that DDB facilitates lysosomal acidification by promoting lysosomal biogenesis.

### 3.6 DDB Promoted the Nuclear Translocation of TFEB

**FIGURE 6:**
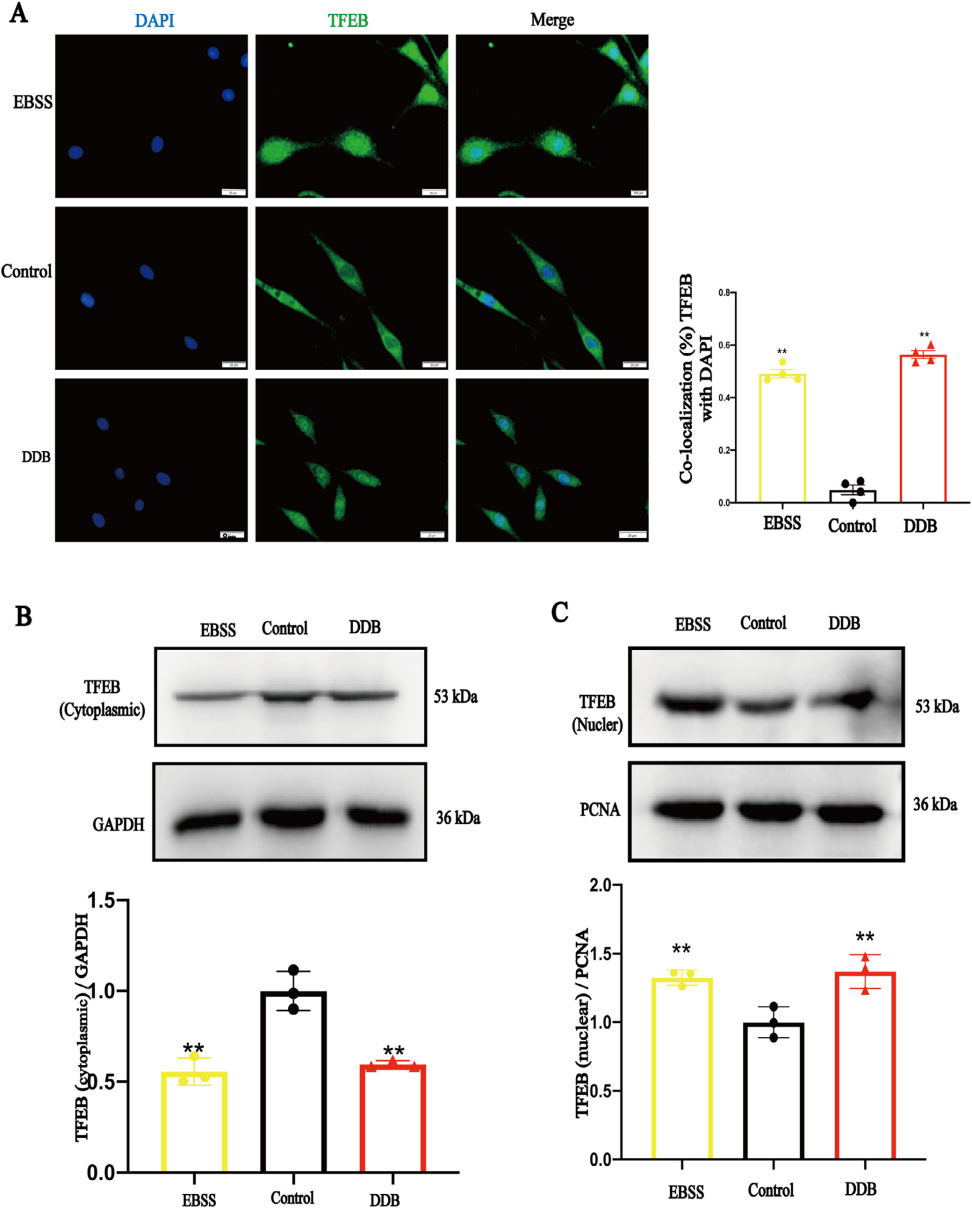
The effect of DDB on TFEB. (A) Representative microscopic images of TFEB with the cytosolic dye DAPI are shown (scale bar = 20 μm, $\bar{x}$ ± SD, *n* = 4). (B) Quantitative statistics of representative bands and protein expression levels of TFEB plasma proteins ($\bar{x}$ ± SD, *n* = 3); (C) Quantitative statistics of representative bands and protein expression levels of TFEB plasma proteins ($\bar{x}$ ± SD, *n* = 3); **p < 0.01 vs. Control group.

The MiT/TFE family transcription factor (TFEB) is a master regulator for the transcription of genes involved in autophagy and lysosomal biogenesis [31]. It is activated by dephosphorylation and is translocated to the nucleus where it upregulates the transcription of its target genes, such as v‐ATPase [32]. Immunofluorescence was used to observe the co‐localization of TFEB with the nuclear dye DAPI (Figure 6A). The results showed that the co‐localization of TFEB with DAPI was higher in the DDB group compared to the control group. The nuclear translocation of TFEB was also analyzed by WB detection of nuclear and plasma protein levels (Figure 6B,C), and the results showed that compared with the control group, the plasma TFEB protein level was significantly decreased and the nuclear protein level was significantly increased in the EBSS and DDB groups. These results suggest that DDB promotes the nuclear transcription of TFEB.

### 3.7 DDB Promoted the Separation of mTOR From Lysosome

**FIGURE 7:**
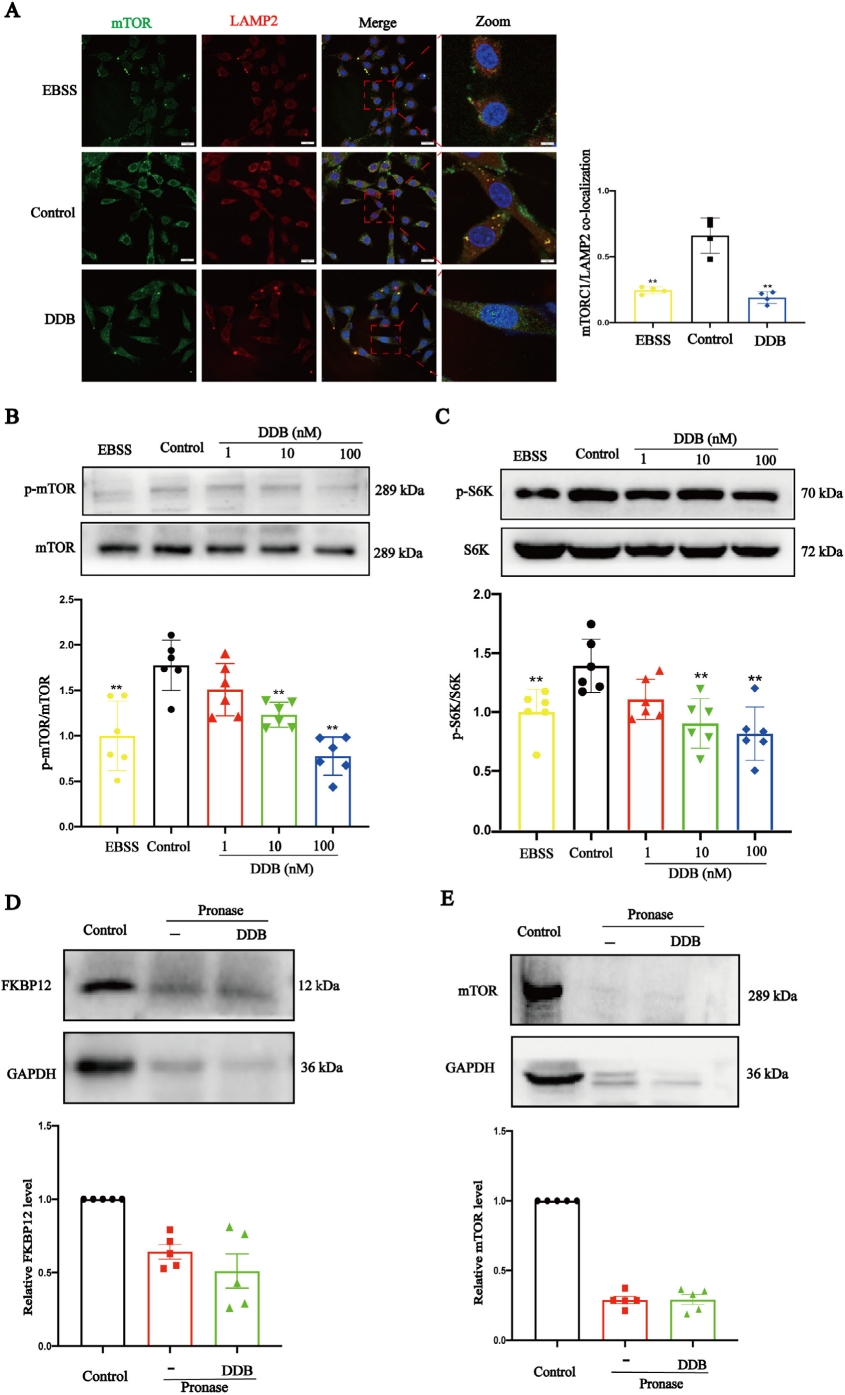
The effect of DDB on mTOR. (A) Co‐localization of mTOR and lysosome (scale bar = 20 μm). ($\bar{x}$ ± SD, *n* = 4). (B and C) Representative bands and quantitative statistics of protein expression levels for p‐mTOR (ser2448), p‐S6K (pT389) are shown. ($\bar{x}$ ± SD, *n* = 5). (D and E) DARTS samples with WB for FKBP12 and mTOR protein quantified relative to its Control. ($\bar{x}$ ± SD, *n* = 5), *p < 0.05, **p < 0.01 vs. Control group.

Mammalian target of rapamycin (mTOR) Complex 1 (mTORC1) is an important and highly conserved regulator of cell growth, which is recruited to lysosomes and thus activated under nutrient‐sufficient conditions [33]. In its inactivated state, mTORC1 separates from lysosomes and promotes nuclear translocation and activation of TFEB [34, 35, 36]. The co‐localization of mTOR and labeled lysosomal marker protein (LAMP2) was observed by immunofluorescence staining (Figure 7A). The results showed that the co‐localization of mTORC1 with LAMP2 in the DDB group was significantly lower than that in the control group and similar to that in the EBSS group, which showed that DDB promoted the separation of mTOR and lysosomes.

mTORC1 is inactivated after detachment from the lysosome [37]; we evaluated levels of phospho‐mTOR (p‐mTOR) and phospho‐p70S6K (p‐p70S6K) by WB. As the concentration of DDB increased, the levels of p‐mTOR and p‐p70S6K decreased (Figure 7B,C). In addition, we also tested the binding of DDB to two current drug targets that inhibit mTOR activity (mTOR and FKBP12) by DARTS and found that DDB did not bind to either of them (Figure 7D,E). These results provide further evidence that DDB inhibits the recruitment of mTORC1 to lysosomes.

### 3.8 DDB Targets ATP6V1A in PC12 Cells

**FIGURE 8:**
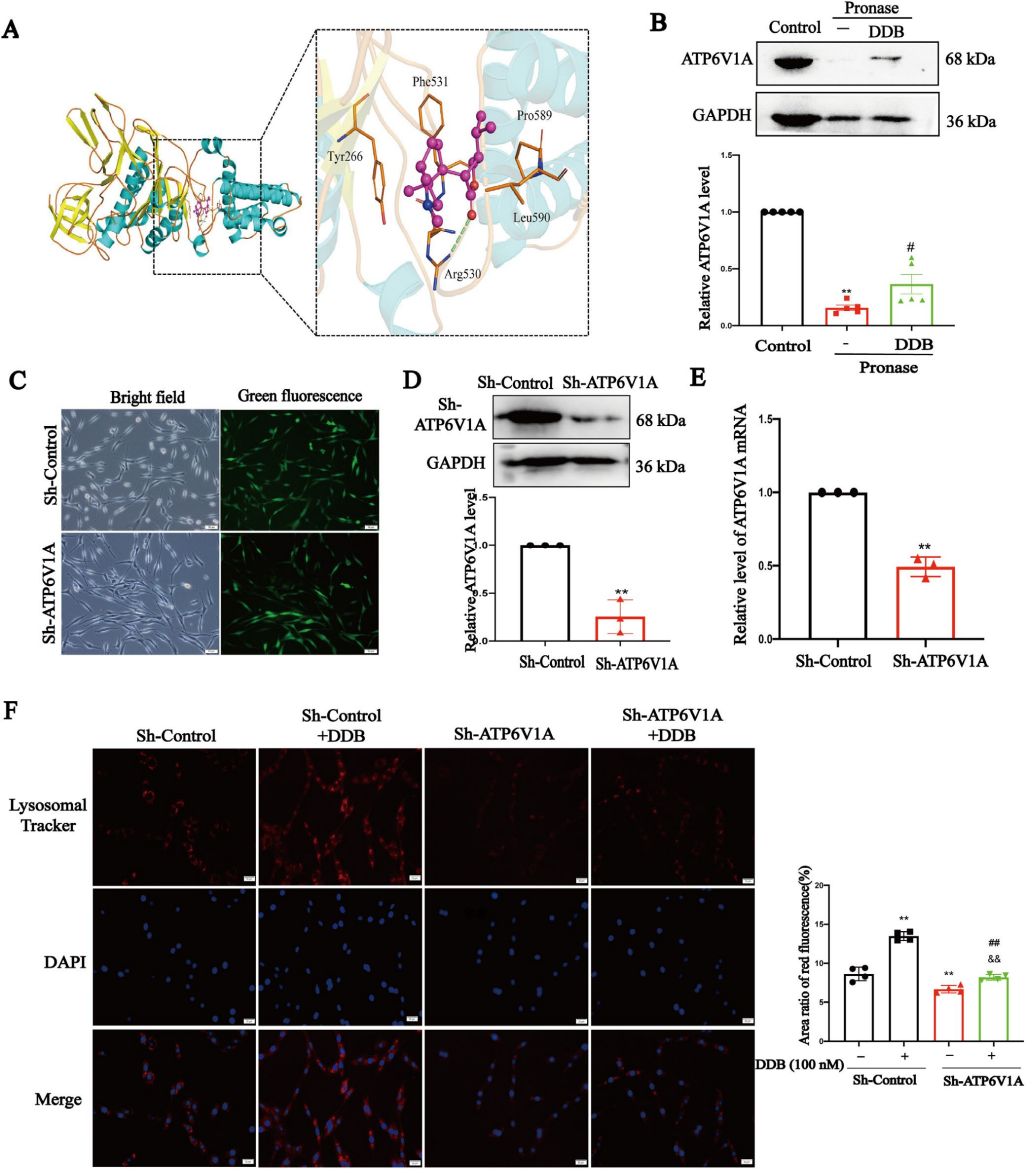
DDB targets ATP6V1A. (A) Molecular docking detection of DDB binding to ATP6V1A, the binding energy of DDB to ATP6V1A is −5.8 kcal/mol; (B) DARTS samples with WB for ATP6V1A and GAPDH quantified relative to its Control. (C) Representative pictures of GFP‐positive PC12 cells (scale = 20 μm); (D) Comparative statistical plots of ATP6V1A mRNA levels in ATP6V1A PC12 cell line; (E) Comparative statistical plots of ATP6V1A protein levels in ATP6V1A‐shRNA PC12 cell lines; (F) The effect of DDB on lysosomal acidification in Sh‐ATP6V1A PC12 cells and Sh‐Control PC12 cells. ($\bar{x}$± SD, *n* = 5), *p < 0.05, **p < 0.01 vs. Control group, ^#^ *p* < 0.05, ^##^ *p* < 0.01 vs. Sh‐Control+100 nM group, ^&^ *p* < 0.05, ^&&^ *p* < 0.01 vs. Sh‐ATP6V1A group.

It has been reported that small molecule drugs can promote the separation of mTOR and lysosome through targeted binding with ATP6V1A, which provides a new possible drug target for this experiment [38]. Therefore, the binding between ATP6V1A protein crystal structure (PDB ID: 6WLZ) and DDB (442523) was simulated by computer molecular docking method. The results showed that DDB could bind to ATP6V1A subunit with binding energy of −5.8 kcal/mol (Figure 8A). It shows that DDB can directly combine with ATP6V1A and has good binding force. We also used the Darts method to validate the direct interaction between DDB and ATP6V1A. The stability of ATP6V1A was significantly decreased when treated with pronase alone, and ATP6V1A stability was significantly increased after pretreatment with DDB (Figure 8B). Thus, the results showed that DDB could protect the ATP6V1A protein in this band against the digestion of streptomyces protease.

To validate that ATP6V1A is a target for DDB even further, we also used shRNA to knock down ATP6V1A gene expression in the PC12 cells. The constructed Control‐shRNA and ATP6V1A‐shRNA lentiviruses were transfected into PC12 cells, and the transfection results were shown in Figure 8C. WB and PCR were used to detect ATP6V1A protein and mRNA levels. The ATP6V1A mRNA levels of Sh‐ATP6V1A stable strains were 49% of the Sh‐Control group (Figure 8D), and the ATP6V1A protein levels were 25% of the control group (Figure 8E), which indicated that the PC12 cell line with stable expression of Sh‐ATP6V1A had been obtained. Lysosomal acidification was also detected using the LysoTracker Red method. Compared with the Sh‐Control group, the red fluorescence intensity of the Sh‐Controlled + DDB group significantly increased. While the effect of DDB (100 nM) in increasing red fluorescence intensity was significantly inhibited in ATP6V1A‐gene‐silenced PC12 cells (Figure 8F). These results suggest that DDB promotes lysosomal acidification by targeting ATP6V1A.

### 3.9 DNLA Promoted Lysosomal Localization of mTORC1 and TFEB Nuclear Translocation by Targeting ATP6V1A in APP/PS1 Mice

**FIGURE 9:**
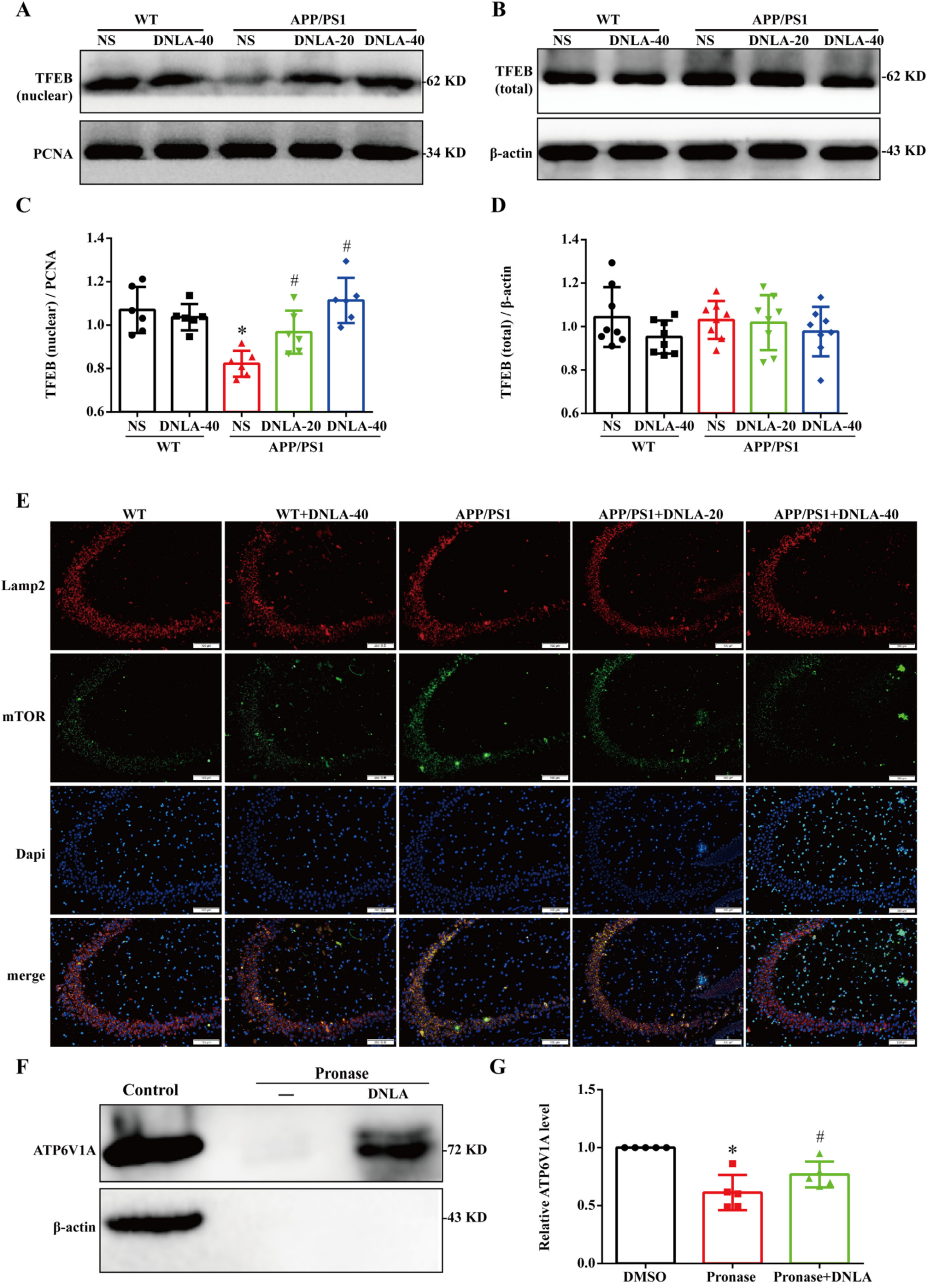
Effects of DNLA on TFEB nuclear translocation in the hippocampus of APP/PS1 mice. (A and B) Representative bands of nucleus TFEB and total TFEB. (C and D) Quantification of protein expression levels of nucleus TFEB and total TFEB. (E) Co‐localization of mTOR and lysosome. (F) Darts samples were detected by WB. (G) Quantitative statistics of relative ATP6V1A protein levels ($\bar{\chi }$ ± SD, *n* = 3), *p < 0.05.

The results of PC12 cells suggest that DDB promotes lysosomal acidification by targeting ATP6V1A and regulating the mTOR/TFEB/v‐ATPase pathway. So we further verified the results in APP/PS1 mice. WB was again used to observe the nuclear translocation of TFEB in the hippocampus of APP/PS1 mice. The results showed that the total expression of TFEB in each group was unchanged; the level of TFEB protein in the nucleus of APP/PS1 mice was significantly lower than that of WT mice, while the level of TFEB protein in the nucleus of the DNLA‐40 group showed no differences to the WT group (Figure 9A–D). The immunofluorescence double labeling was used to observe the co‐localization of mTOR and lysosomal marker Lamp2. The results showed that the fluorescence co‐localization of mTOR and Lamp2 was significantly increased in APP/PS1 mice compared with WT mice, while the fluorescence co‐localization of mTOR and Lamp2 was significantly reduced after DNLA treatment (Figure 9E). Darts assay was used to investigate the potential of DNLA to bind ATP6V1A. As shown in Figure 9F,G, the stability of ATP6V1A was significantly decreased when treated with pronase alone, and ATP6V1A stability was significantly increased after pretreatment with DNLA. Thus, the above results also suggest that DNLA promotes lysosomal acidification by targeting ATP6V1A and regulating the mTOR/TFEB/v‐ATPase pathway.

## 4 Discussion

AD is a central nervous system degenerative disease with progressive cognitive impairment and memory impairment as its main clinical manifestations [1]. Dysfunction of the endolysosome‐autophagy network is an early pathological feature of AD [26, 39]. Here, we found that v‐ATPase was significantly down‐regulated prior to learning and memory impairment. Early DNLA intervention ameliorated the AD pathology in aged APP/PS1 transgenic mice, as evidenced by behavioral tests and detection of senescent neurons. The specific mechanism may be that DNLA binds ATP6V1A, leads to the separation of mTOR from lysosome, in return promotes the nuclear translocation of TFEB, and promotes lysosome biogenesis and lysosome acidification.

APP/PS1 mice are classic AD model mice. APP and PS1 mutations are the cause of autosomal dominant, early‐onset single‐gene AD, leading to significant defects in lysosome function and autophagy [23]. With the aging of APP/PS1 mice, the typical pathological features of AD, such as Aβ plaque deposition, learning and memory dysfunction, and neuron and synaptic loss appeared gradually [22]. To investigate whether v‐ATPase activity is implicated in AD pathology, we first examined the learning and memory ability and the expression of v‐ATPase subunits in 4‐month‐old APP/PS1 mice. The results showed that the 4‐month‐old APP/PS1 mice did not present with learning and memory loss and amyloid plaque deposition. However, the protein expression of ATP6V1A and ATP6V0a1 proteins was decreased in the hippocampus. These results suggest that the observed changes in v‐ATPase are early phenomena in the pathological process of AD.

DNLA is the main active ingredient of DNL. Previous studies have reported that it can improve the learning and memory deficit in APP/PS1 transgenic mice by improving lysosomal acidification [40]. In this study, 4‐month‐old APP/PS1 mice were used to determine whether DNLA can delay the learning and memory impairment by regulating v‐ATPase. Behavioral experiments showed that 5months of DNLA administration delayed the manifestation of learning disabilities in mice. Importantly, the persistence of senescent cells late in life is associated with tissue dysfunction and an increased risk of age‐related disease [19]. In our study, we found that the neurons in the brains of 9‐month‐old APP/PS1 mice did not show irreversible pathological changes such as death and loss, but the number of senescent cells in the brain increased. Prophylactic administration of DNLA could reduce the number of senescent cells. These results suggest that DNLA can reduce the number of senile cells and delay the learning and memory disorders.

To explore the mechanism behind DNLA‐induced delay of neuronal aging, the levels of Aβ_1–42_ and Aβ_1–40_ and the deposition of amyloid protein in the brains of APP/PS1 mice were detected. The results showed that preventive administration of DNLA could significantly reduce the levels of total Aβ_1–42 and_ Aβ_1–40_ in the hippocampus. But the extracellular amyloid plaque deposition in the hippocampus did not decrease, suggesting that DNLA may reduce the intracellular Aβ. Studies have reported that intracellular Aβ is mainly stored in AVs and degraded by the autophagolysosomal system [4, 41]. The decreased lysosomal proteolysis function in the brains of AD patients and animal models leads to significant autophagolysosomal accumulation, which may be one of the explanations for the intracellular Aβ accumulation in APP/PS1 mice. Therefore, we measured Aβ_1–42_ content in lysosomes, and the results suggested that DNLA promotes the degradation of Aβ_1–42_ in lysosomes. The expression levels of autophagic lysosomal pathway proteins LC3 and p62 were further detected by WB; the data showed that DNLA reduced the levels of LC3 and p62. Studies have reported that the impaired autophagy function in AD patients is mainly due to the decreased function of the autophagolysosomal enzyme [26]. Therefore, we detected the co‐localization of M6PR and Cat D. The results showed that DDB promoted the separation of M6PR and Cat D, thereby indicating that DNLA promotes the increase of lysosomal acidity. Importantly, v‐ATPase is the main channel protein regulating lysosome acidification. We further detected the protein expression of v‐ATPase subunits, and the results showed that DNLA upregulates the protein expression of ATP6V1A and ATP6V0a1 in the hippocampus of APP/PS1 mice. These results suggest that DNLA may promote lysosome acidification by promoting the protein expression of v‐ATPase subunits, leading to improved autophagy flow and increased intracellular Aβ degradation.

DDB is the main active ingredient in DNLA, and the content of DDB accounts for 93.1% of DNLA in this study. Since DDB is similar to DNLA in increasing the level of ATP6V1A protein, we used DDB to further analyze the specific mechanism of its promotion of lysosome acidification. To explore whether DDB induced lysosomal acidification, PC12 cells were used to examine the acidification of lysosomes and the protein levels of v‐ATPase subunits. PC12 cells are derived from rat pheochromocytoma and are commonly used neuron‐like cell lines with a single cell component and can be cultured by passage. So PC12 cells were used as tools to analyze the specific molecular mechanisms in detail. The results suggested that DDB promotes lysosomal acidification and the protein levels of v‐ATPase subunits, and we also observed similar results in primary hippocampal neurons and HT22 cells. We also tested the effect of DDB on v‐ATPase activity by ELISA and found that DDB significantly increased v‐ATPase activity. RT‐PCR was used to examine the levels of lysosomal‐related genes, and the results showed that DDB upregulated the transcript levels of these genes, which suggests that DDB facilitates lysosomal acidification by promoting lysosomal biogenesis.

TFEB is believed to be a major regulator of the autophagy and lysosomal pathways, responding to lysosomal pH and content through the lysosomal trophic induction (LYNUS) mechanism (consisting of v‐ATPase, 2Rag‐GTPases, and regulators) and recruitment of mTORC1 and conducting cytoplasmic to nuclear transport. Inside the nucleus, TFEB promotes the transcription of its target genes involved in autophagy, lysosomal biogenesis [42]. At present, the confirmed regulated genes include but are not limited to 73 autophagy lysosomal pathway‐related genes, 23 lysosomal hydrolase and helper protein genes, 9 lysosomal‐associated membrane protein genes, and 14 v‐ATPase subunit genes []. Therefore, WB and immunofluorescence were used to quantify the nuclear translocation of TFEB. The results showed that DDB promoted TFEB entry into the nucleus. mTOR is a key upstream regulator of the nucleation of TFEB [43], mTORC1 (mammalian TOR complex 1) and mTORC2 are present in cells. mTORC1 is primarily localized to the lysosome, and when mTORC1 is detached from the lysosome and inactivated, its downstream substrate TFEB translocates to the nucleus [44, 45]. Fluorescence double labeling was used to observe the co‐localization of mTOR and lysosomes. The results showed that DDB could promote the separation of mTOR and lysosomes. We get the same results in 9‐month APP/PS1 mice (Figure 8A–F). So far, we have found that DDB can promote TFEB nuclear translocation by promoting the separation of mTORC1 from lysosome, which in turn promotes lysosome acidification. So how does DDB work? It has been reported that small drugs can bind with ATP6V1A, leading to changes in the spatial structure of v‐ATPase, leading to an effect on the localization of mTOR in the lysosome and subsequent nucleation of TFEB [14, 38]. Therefore, we used the computer molecular docking method to simulate the binding between ATP6V1A and DDB. The results showed that DDB can be directly combined with ATP6V1A and has a good binding force. Darts is a technology that can quickly and directly identify potential target proteins of small molecule drugs. The principle is that after small molecule drugs bind to target proteins, the target protein undergoes conformational changes, making it more resistant to protease‐induced degradation, and then maintaining the activity and function of the target proteins [46]. Darts results (Figures 8B and 9F,G) also showed that DDB can bind to ATP6V1A. After ATP6V1A silencing, the effect of DDB promoting lysosome acidification also greatly reduced. These data indicated that DDB can target ATP6V1A, promote the separation of mTOR from lysosome, promote nuclear translocation of TFEB, and promote lysosomal biogenesis. In this study, PC12 cells were only used as tool cells to elucidate the specific molecular mechanism of DDB promoting lysosomal acidification, and our further verification of this mechanism will be carried out in AD‐related cell models in the follow‐up study.

**FIGURE 10:**
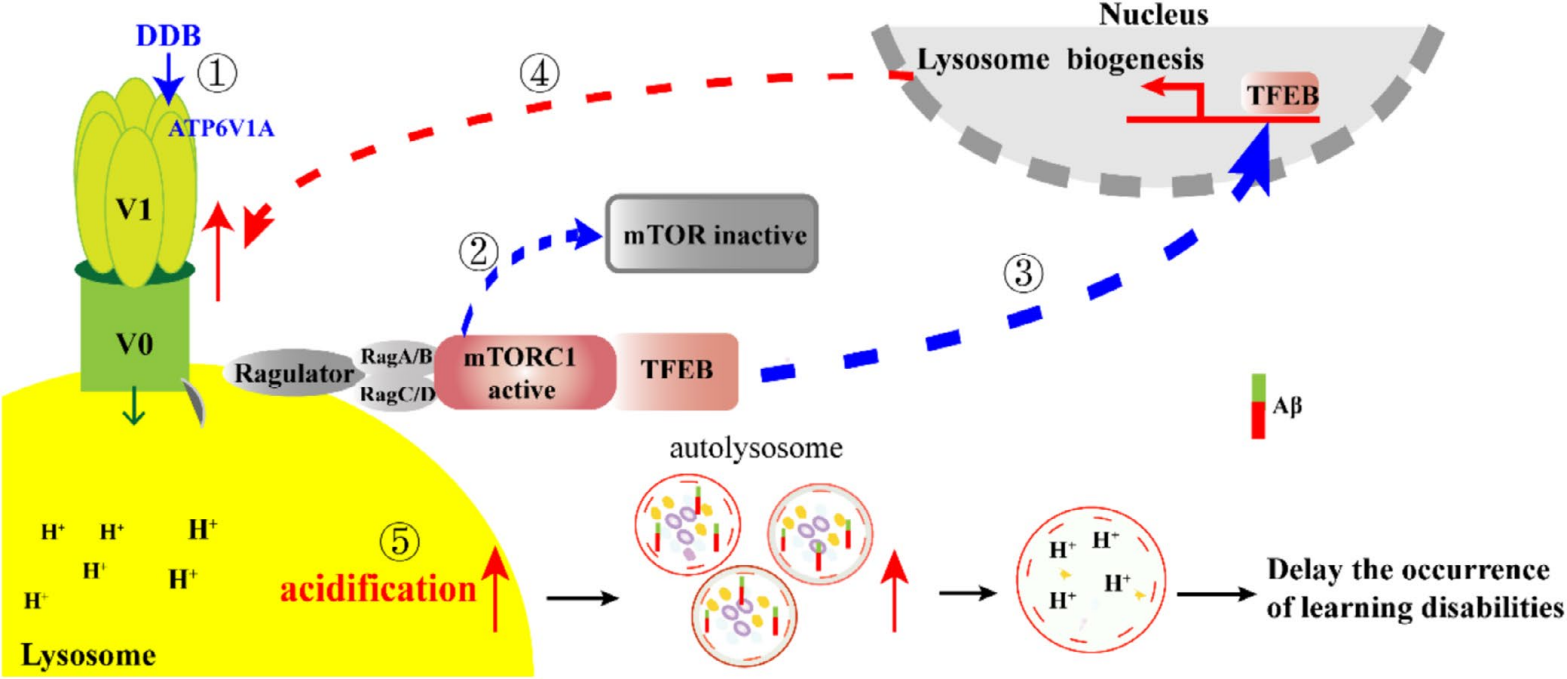
The schematic presentation elucidating proposed mechanisms that DNLA delays the learning and memory impairment. ① DDB may target ATP6V1A. ② DDB promotes the separation of mTORC1 from lysosomes and inactivates mTOR. ③ DDB increases the nuclear translocation of TFEB and promotes lysosomal biogenesis. ④ DDB up‐regulates the protein level of v‐ATPase. ⑤ DDB promotes lysosome acidification, promotes the clearance of Aβ and delays the occurrence of learning disabilities.

In conclusion, we report that DNLA may delay the occurrence and development of AD by targeting ATP6V1A and mediating the mTOR/TFEB/v‐ATPase Signaling Pathway (Figure 10).

## Author Contributions

J.N. contributed to the design of the study, supervision, funds, and the manuscript revision. Y.W. performed the animal experiments, statistical analysis, and wrote the first draft of the manuscript. X.L. contributed to the cell experiments. G.L. was responsible for the supplementary experiment of the manuscript. Q.L. contributed to the mice raising, Morris water maze, and nesting experiments. B.G. contributed to the WB experiment. L.L. contributed to the design of the study, supervision, and funds. All authors contributed to manuscript revision, read and approved the submitted version.

## Ethics Statement

The animal study was reviewed and approved by the experimental Animal Ethics Committee of Zunyi Medical University.

## Conflicts of Interest

The authors declare no conflicts of interest.

## Supporting information


Figure S1

Appendix S1

## Data Availability

The data generated from this study are available upon request from the corresponding authors.
