# Supplementary material for: DNLA Delayed the Appearance of Learning and Memory Impairment of APP/PS1 Mice: Involvement of mTOR/TFEB/v‐ATPase Signaling Pathway
Source: CNS Neurosci Ther. 2025 Mar 6;31(3):e70300. doi: 10.1111/cns.70300 (PMC11883424; doi:10.1111/cns.70300)
Supplement: Supplementary file 1 — Figure S1 [file CNS-31-e70300-s002.docx]

**Supplementary figures**


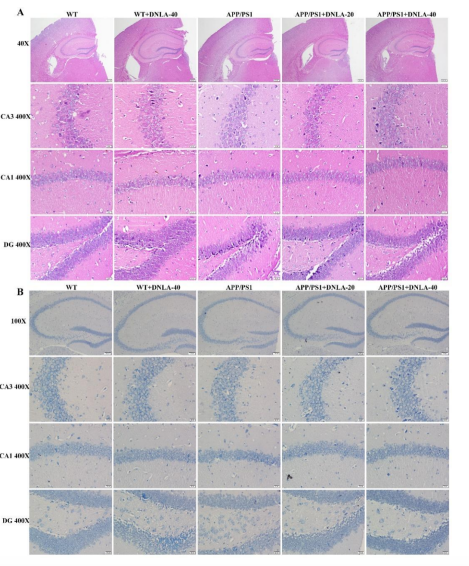


**Fig.S1 Pathological staining of hippocampal neurons of APP/PS1 mice.** (A) Representative images of HE staining. (B) Representative image of nissl staining. (n=3, scale=200 μm, 20 μm)

**
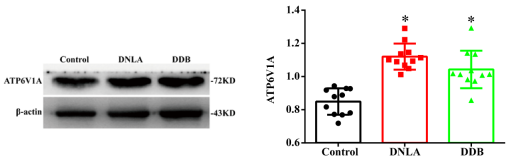
**

**Fig.S2 The effect of DNLA and DDB on ATP6V1A in HT22 cells.** Representative bands and quantification of protein expression level of ATP6V1A. (‾χ ± SD, n = 3), ^*^*P*< 0.05.


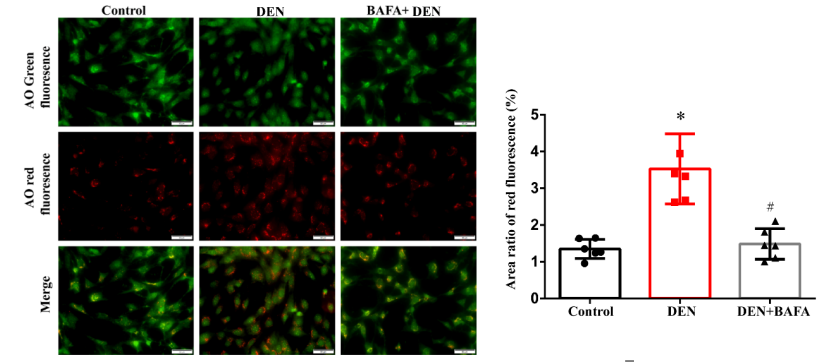


**Fig.S3 The effect of DNLA on lysosomal acidification in HT22 cells.** (‾χ±SD, n=3), * P<0.05 vs. control group, ^#^*P* <0.05 vs. DNLA group.
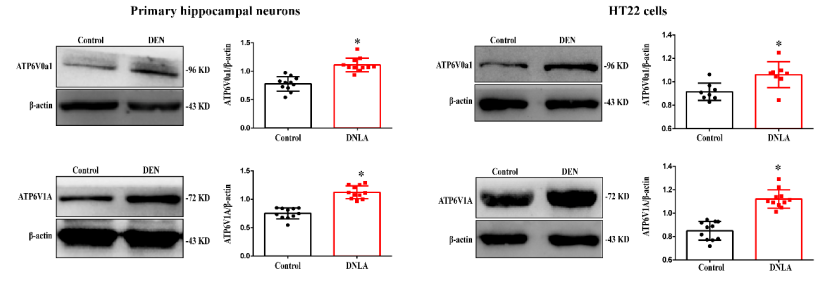


**Fig.S4 The effect of DNLA on the expression of v-ATPase subunits in Primary hippocampal neurons and HT22 cells.** ( ‾χ±SD,n=6), **P* <0.05 vs. control group.


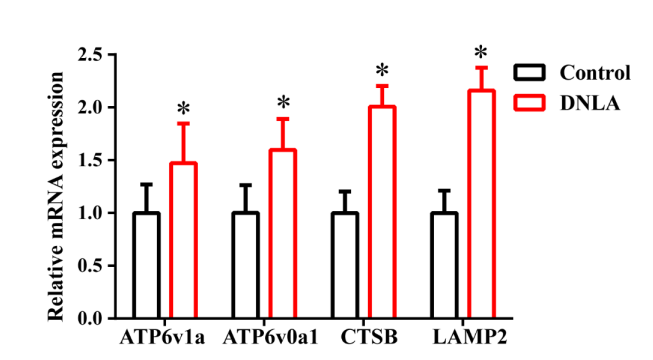


**Fig.S5 The effect of DNLA on the mRNA expression of v-ATPase subunits in HT22 cells.** The mRNA expression of ATP6V1A, ATP6V0a1, CTSB and LAMP2 was detected by RT-PCR assay. (^*^*P* <0.05 *vs.* Control group, $\bar{x}$±SD, n=3)
