# Supplementary material for: DNLA Delayed the Appearance of Learning and Memory Impairment of APP/PS1 Mice: Involvement of mTOR/TFEB/v‐ATPase Signaling Pathway
Source: CNS Neurosci Ther. 2025 Mar 6;31(3):e70300. doi: 10.1111/cns.70300 (PMC11883424; doi:10.1111/cns.70300)
Supplement: Supplementary file 2 — Appendix S1 [file CNS-31-e70300-s001.docx]

The original gels/blots

Fig.1E n=6


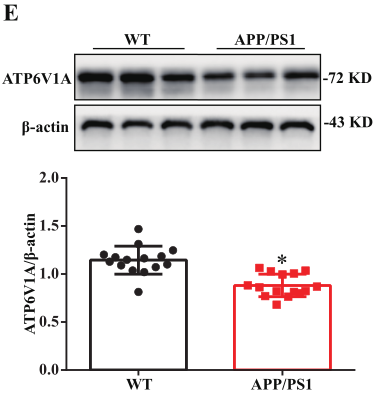


ATP6V1A 72KD


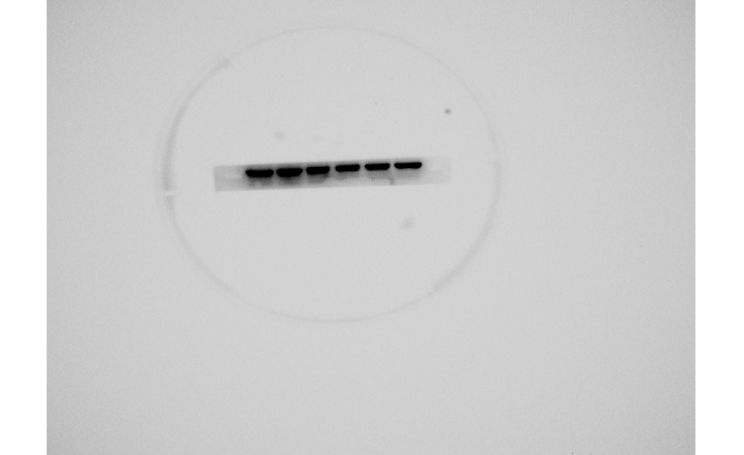

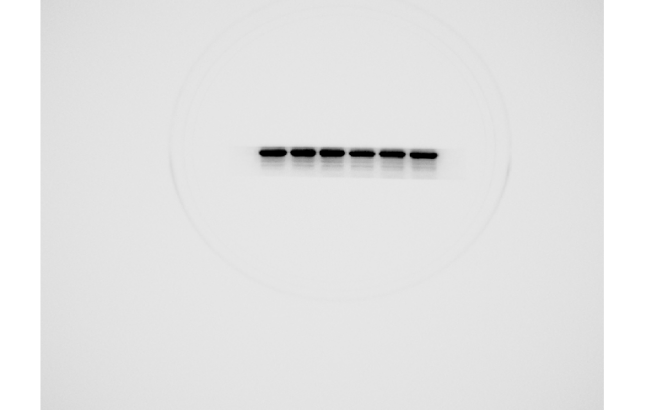

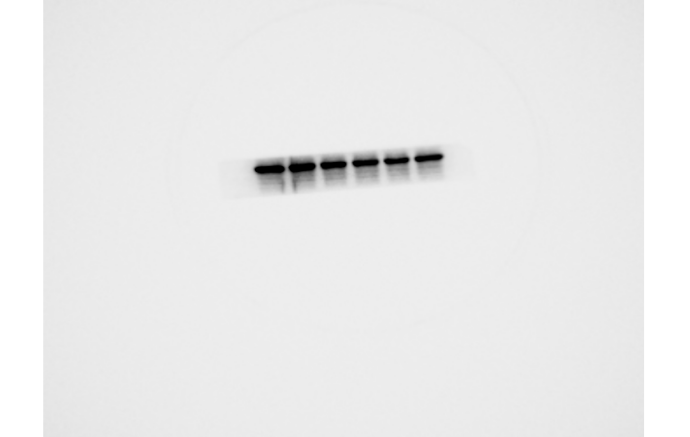


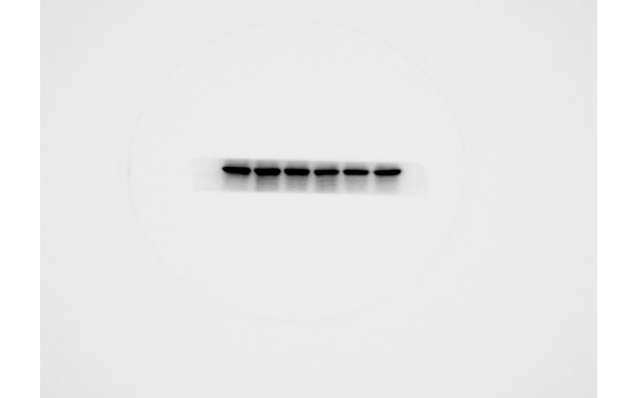

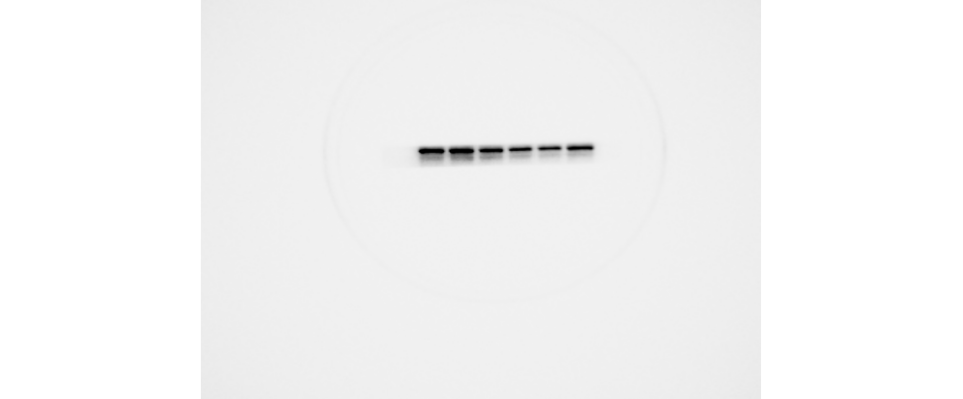


β-actin 43KD


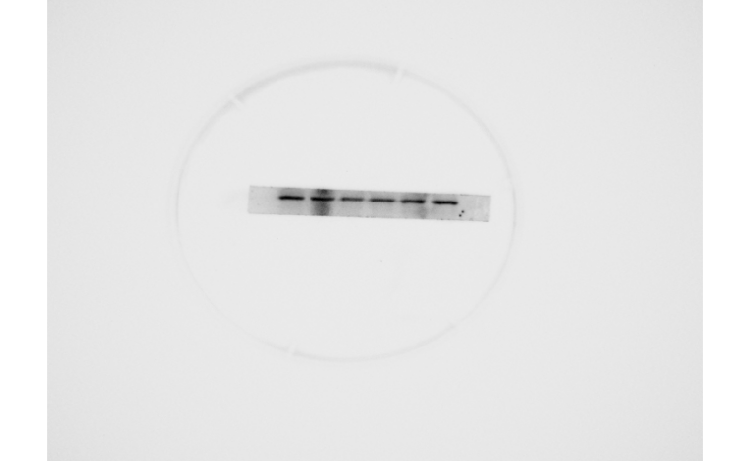

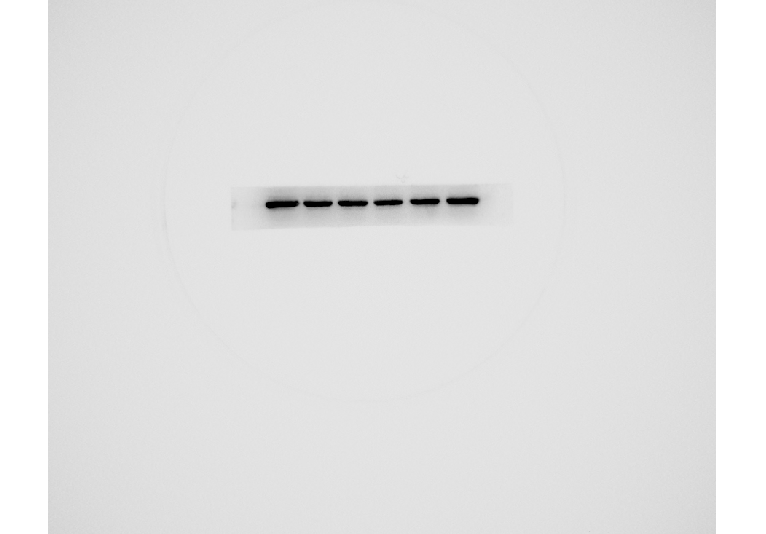

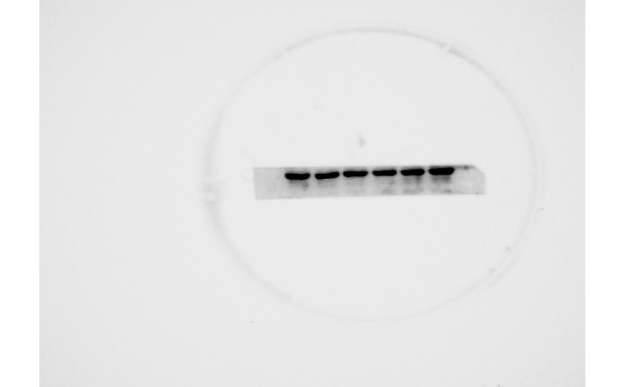


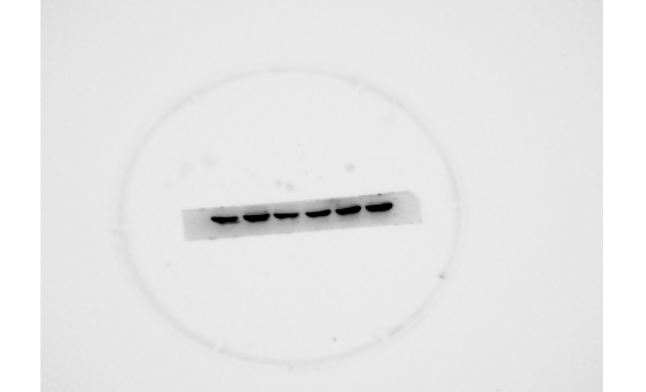

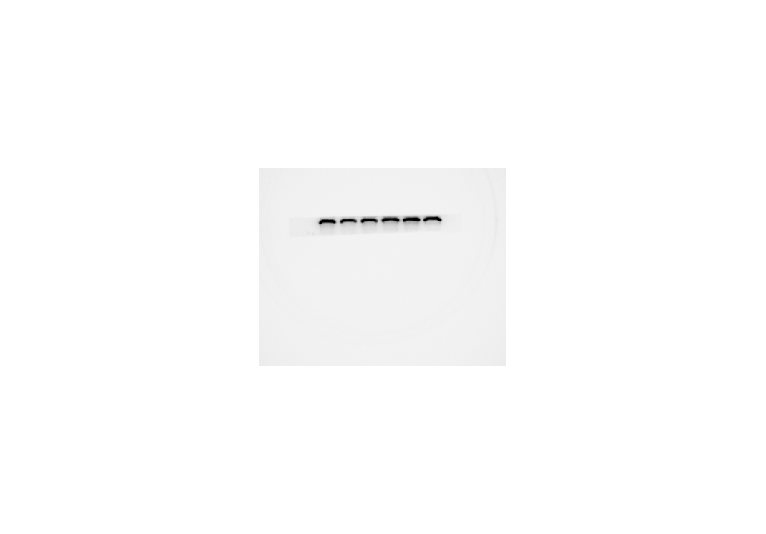


Fig.1F n=6


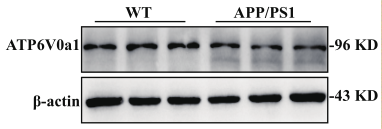


ATP6V0a1 96KD


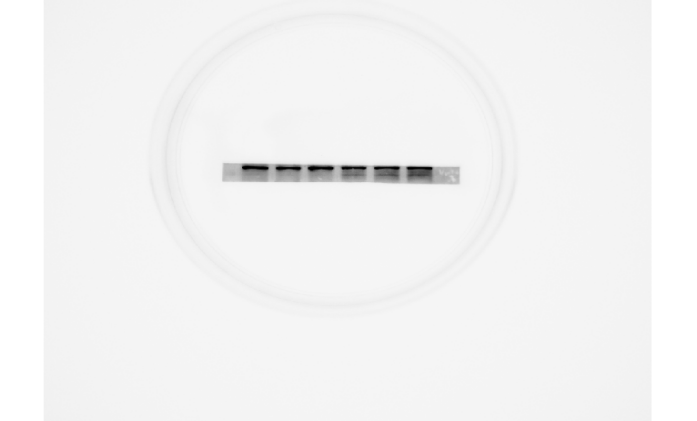

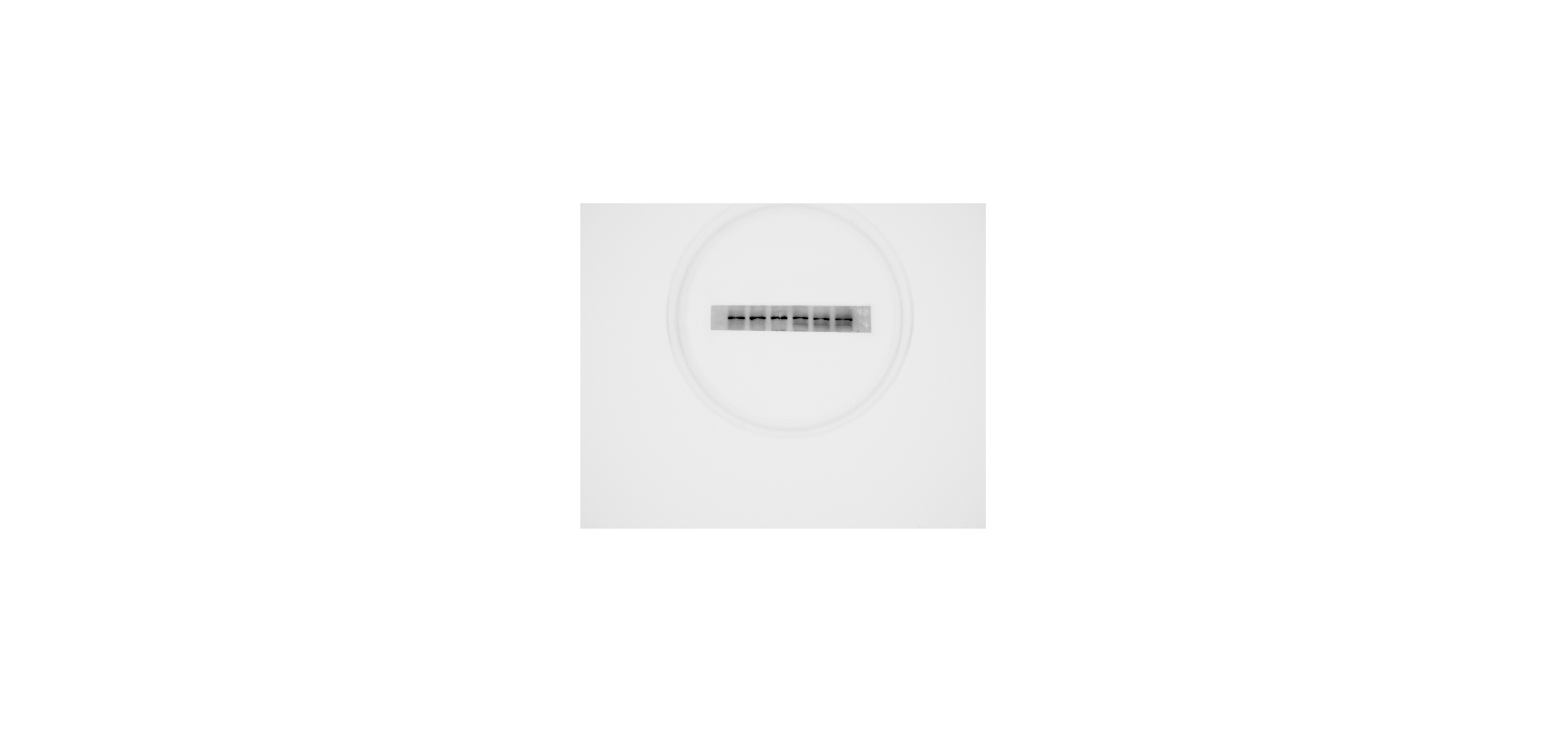

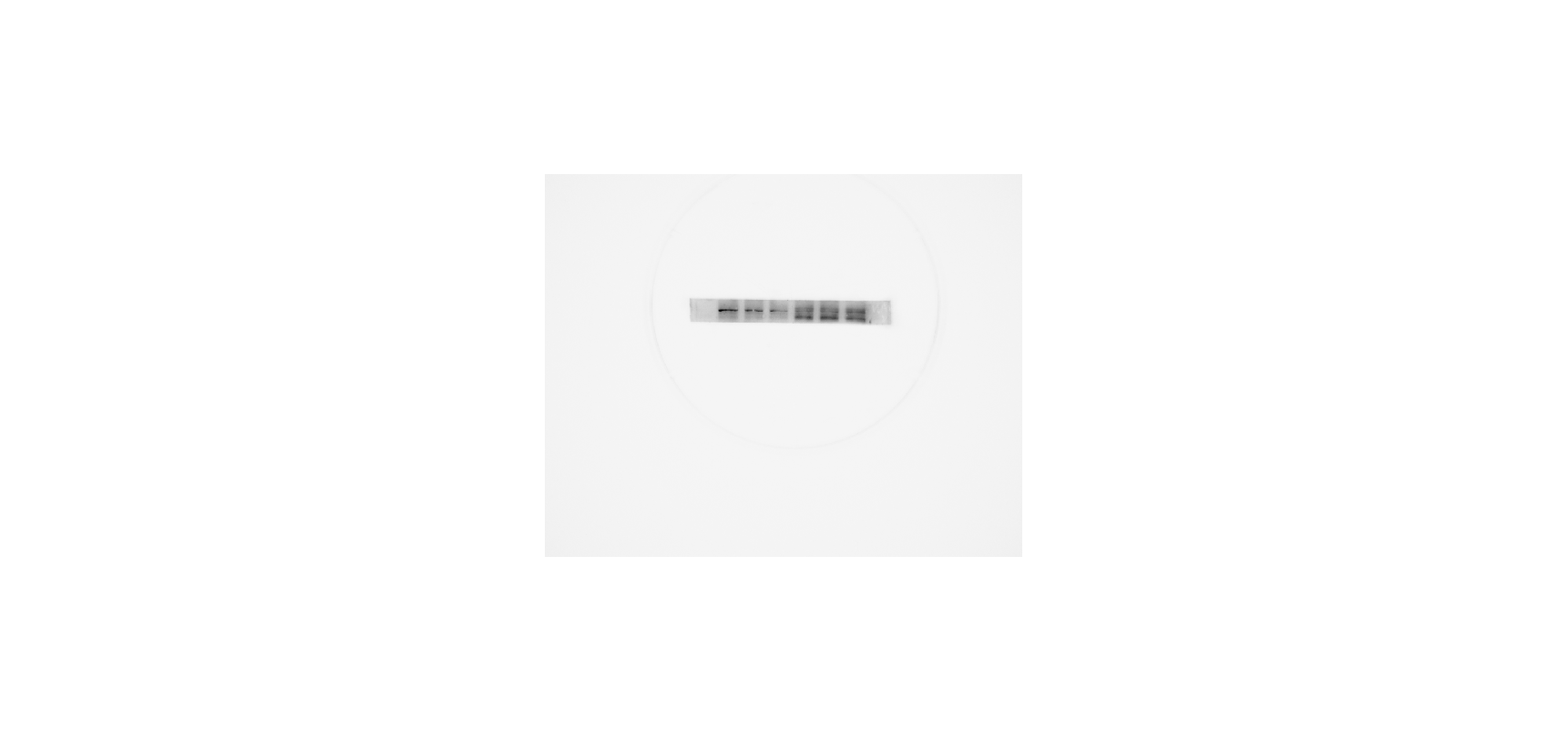


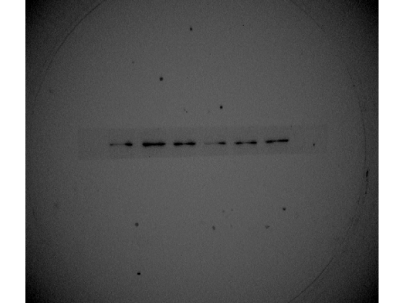

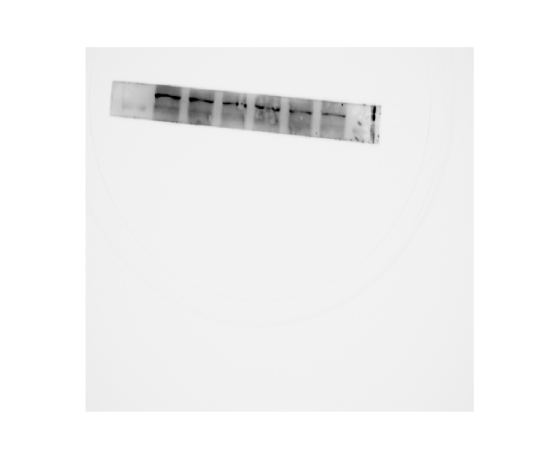

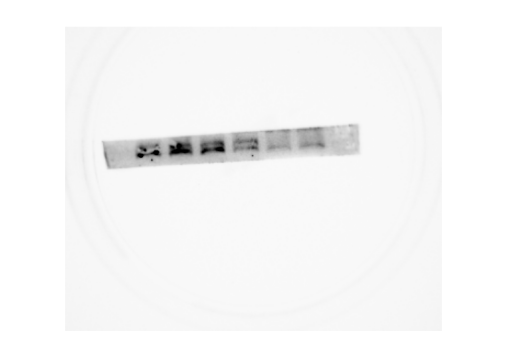


β-actin 43KD


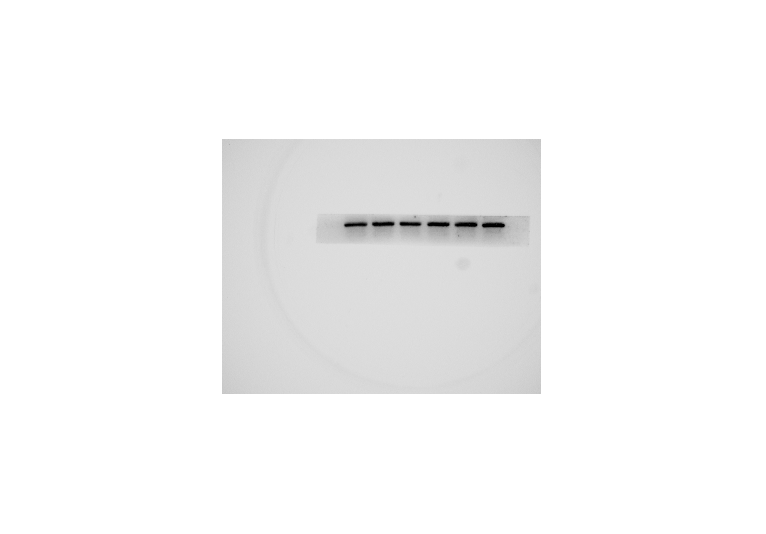

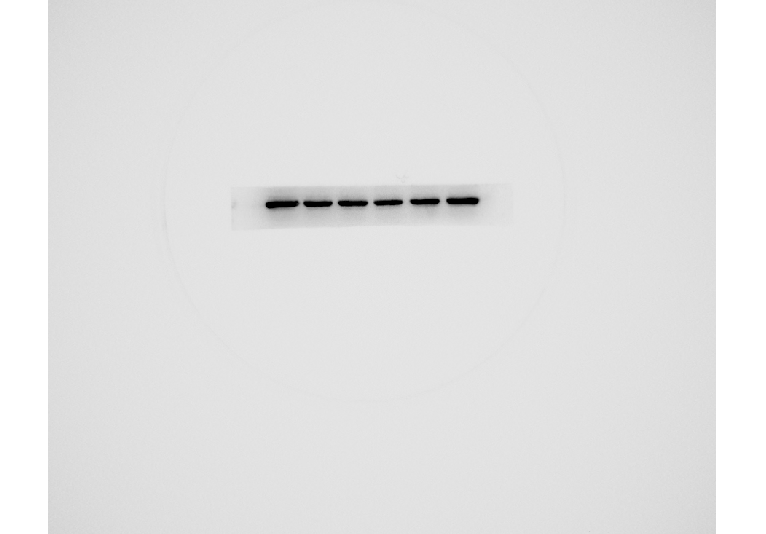

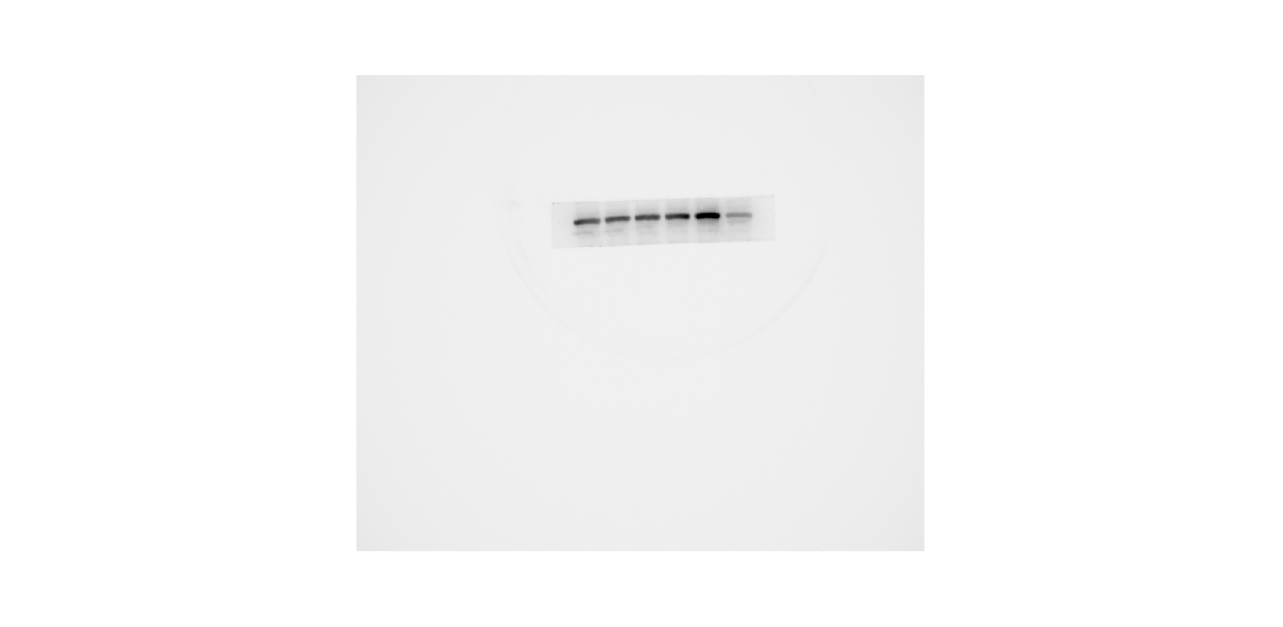


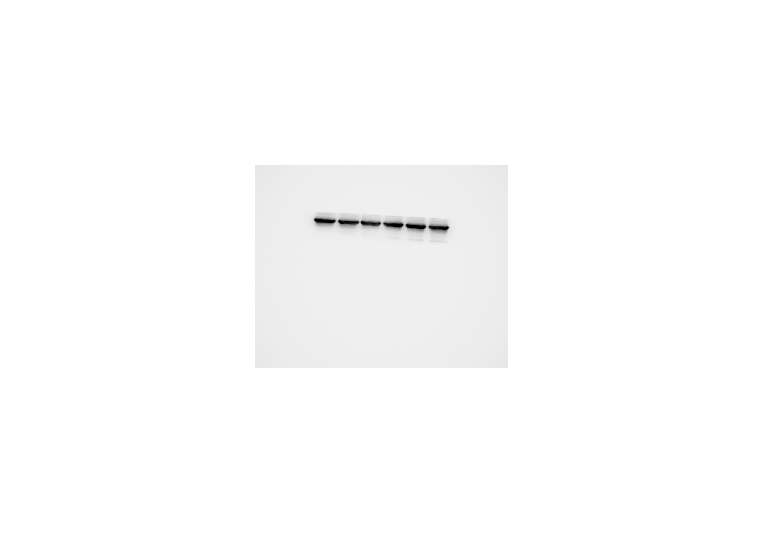

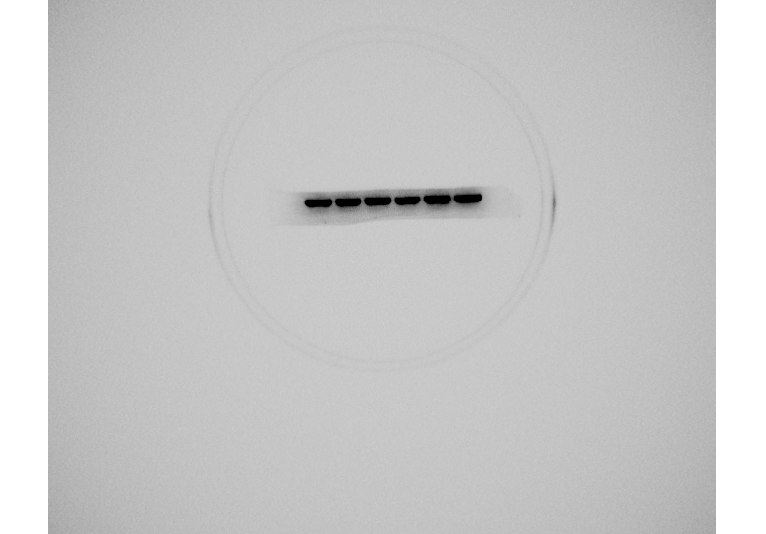

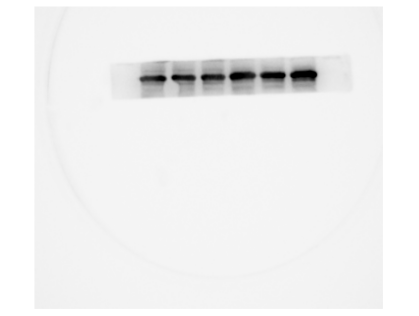


Fig.3C, D and E n=6


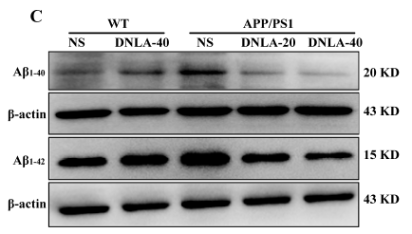


Aβ_1-40_  40KD


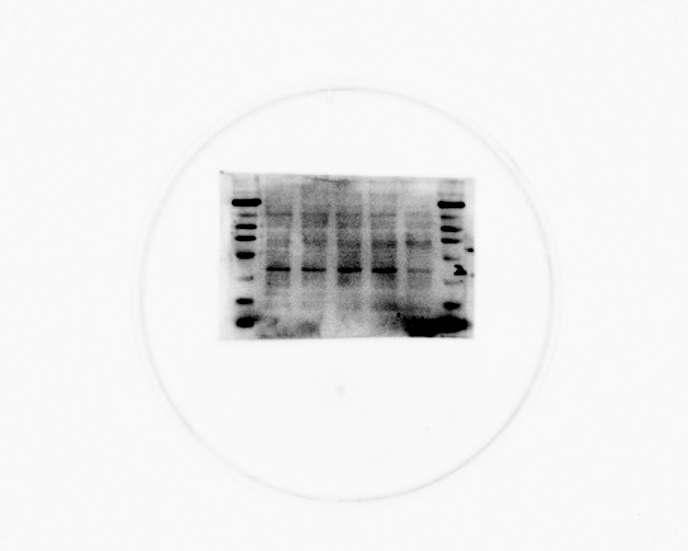

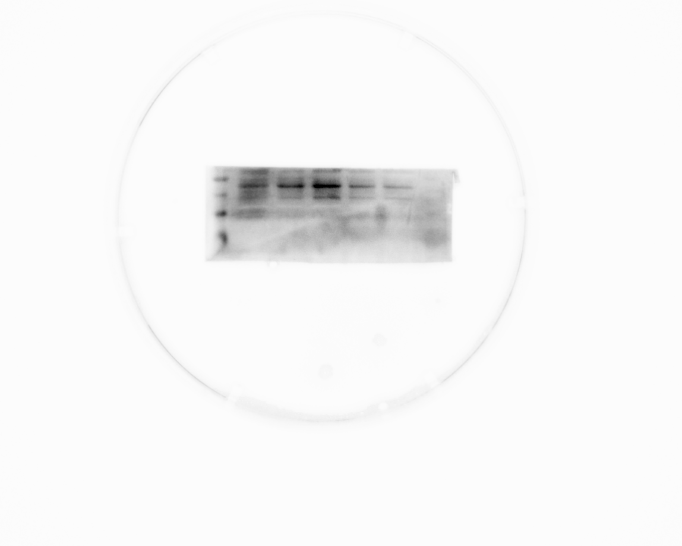

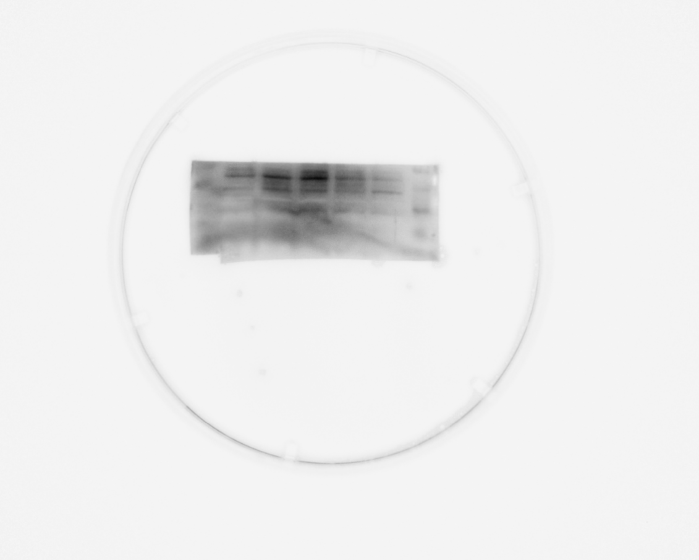


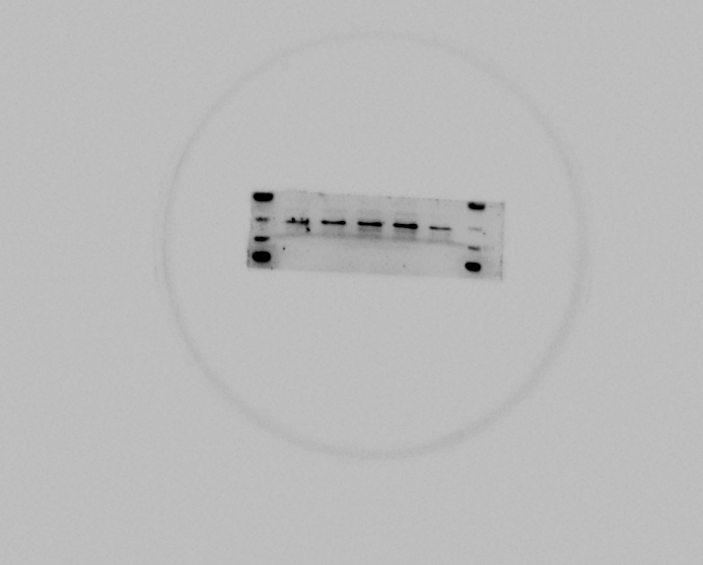

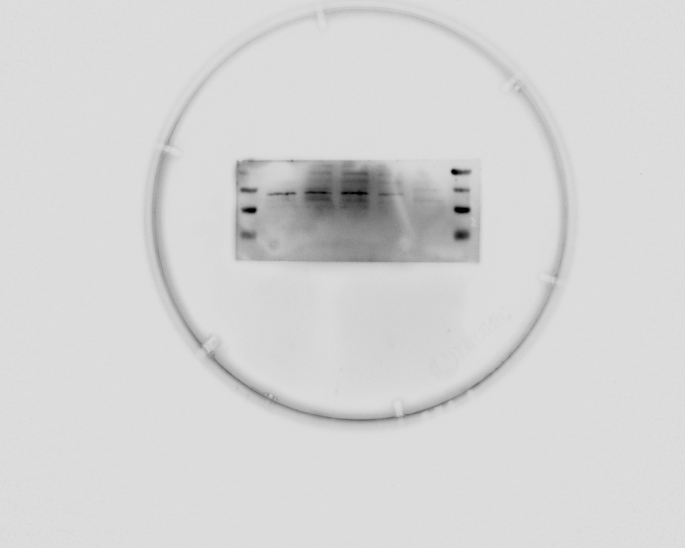

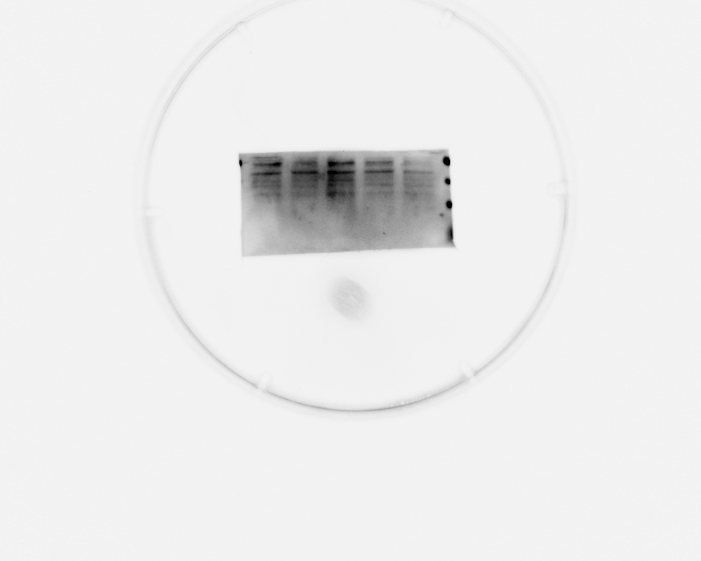


GAPDH 34KD


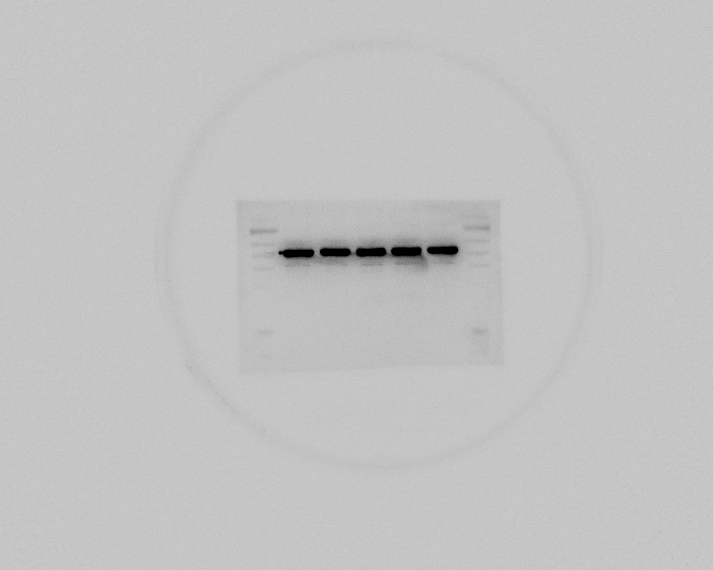

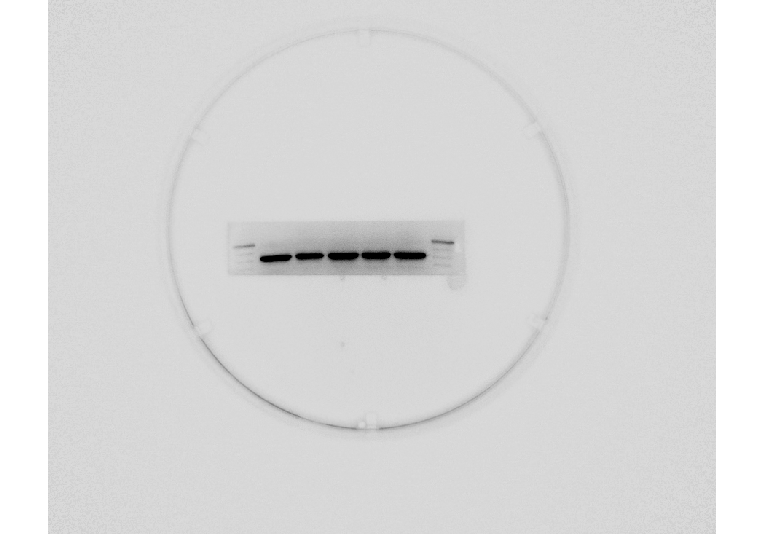

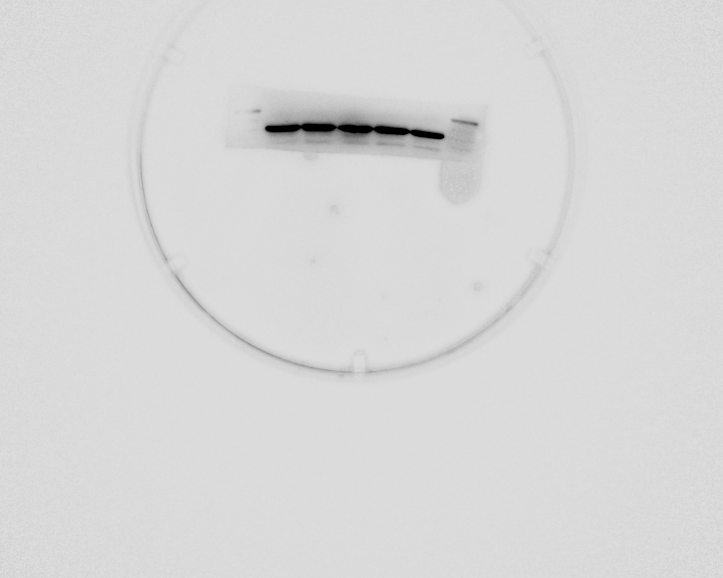


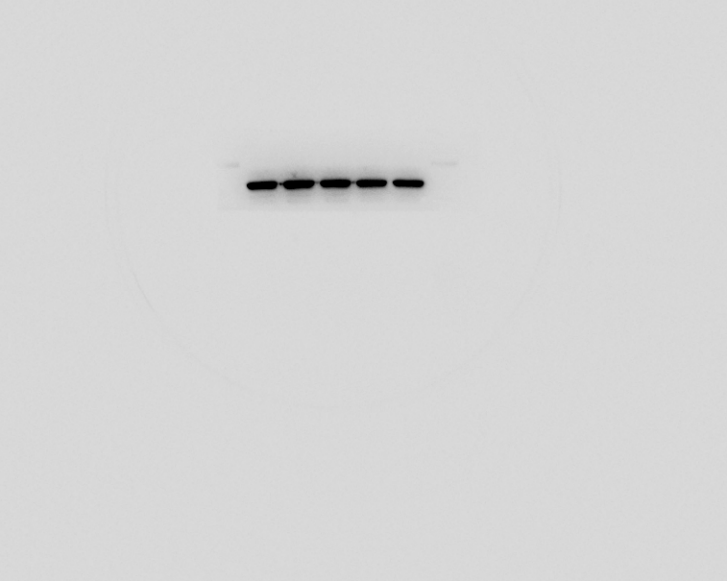

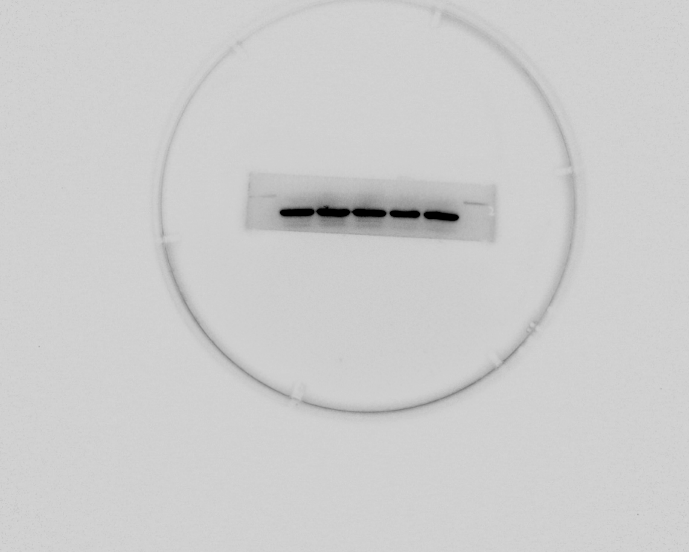

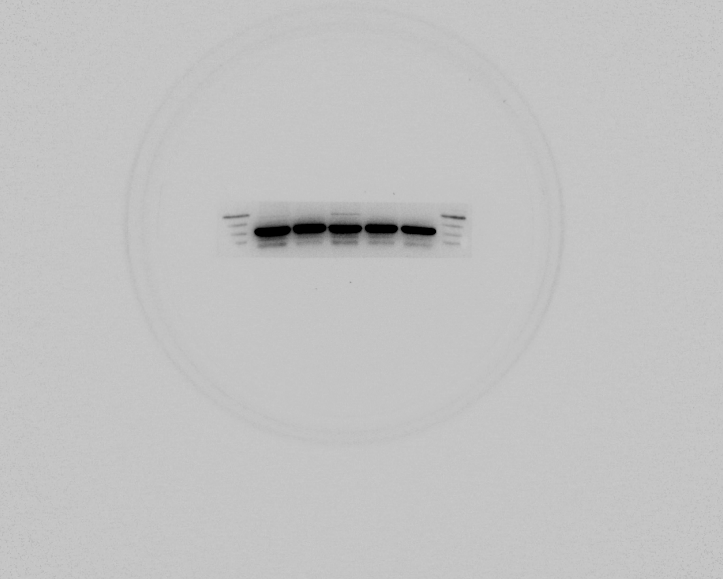


Fig.3C and 3E n=6

Aβ_1-42_  12KD


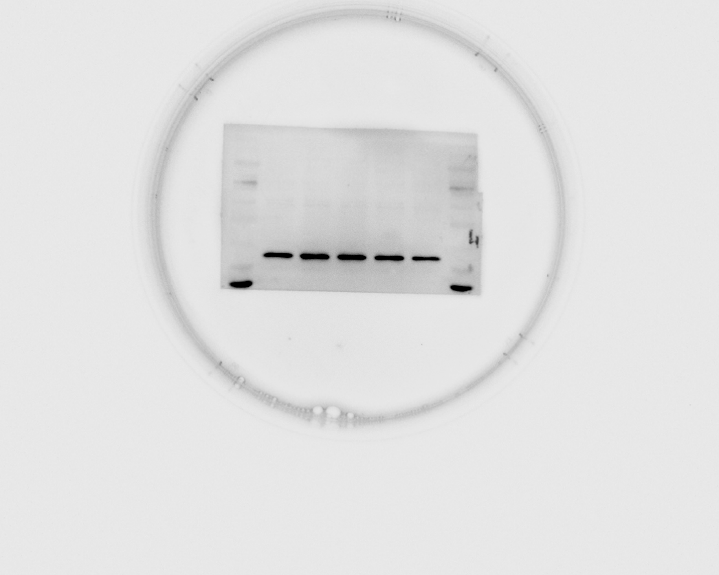

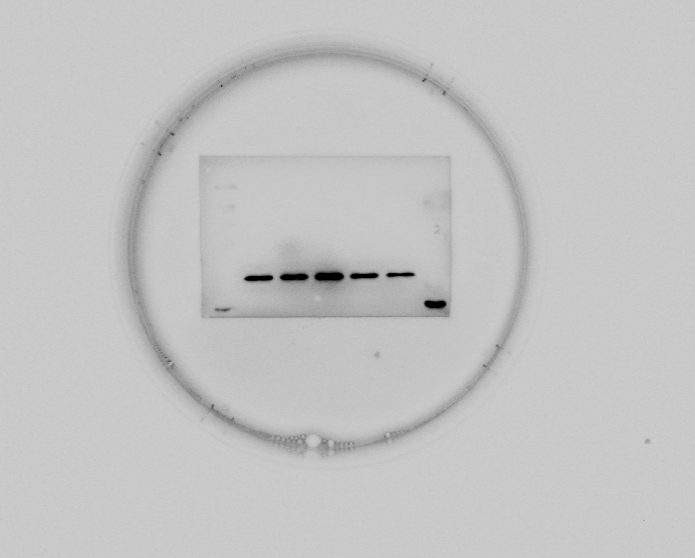

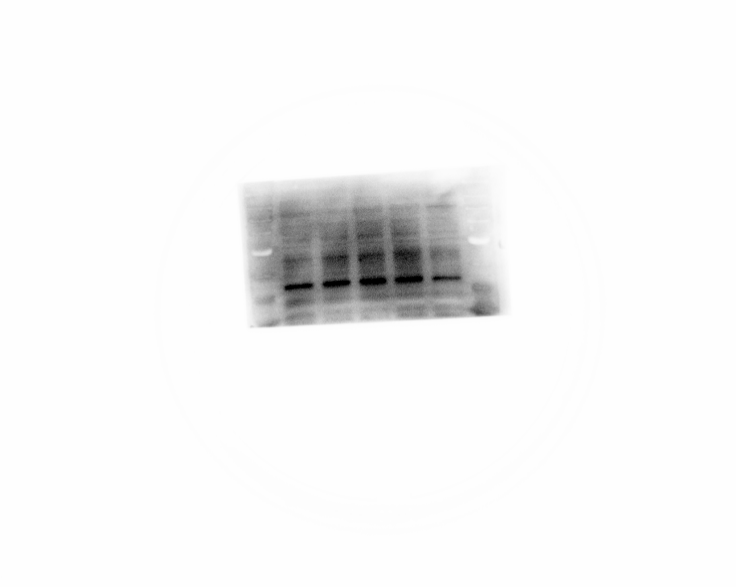


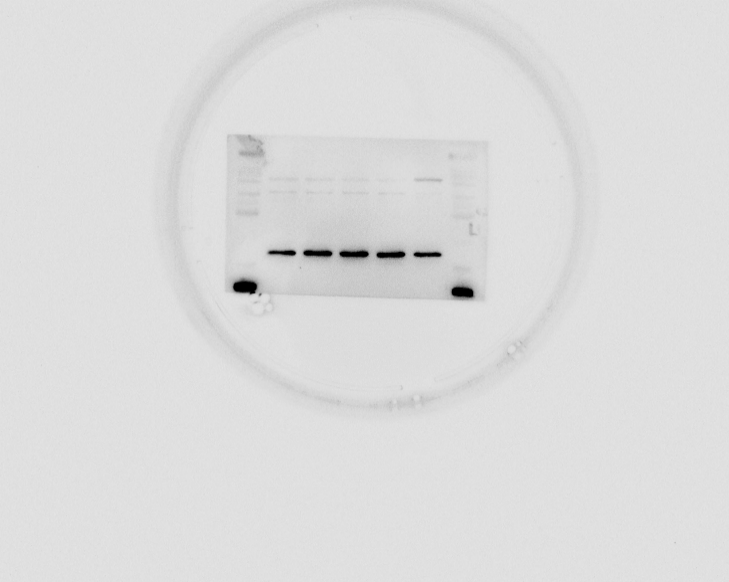

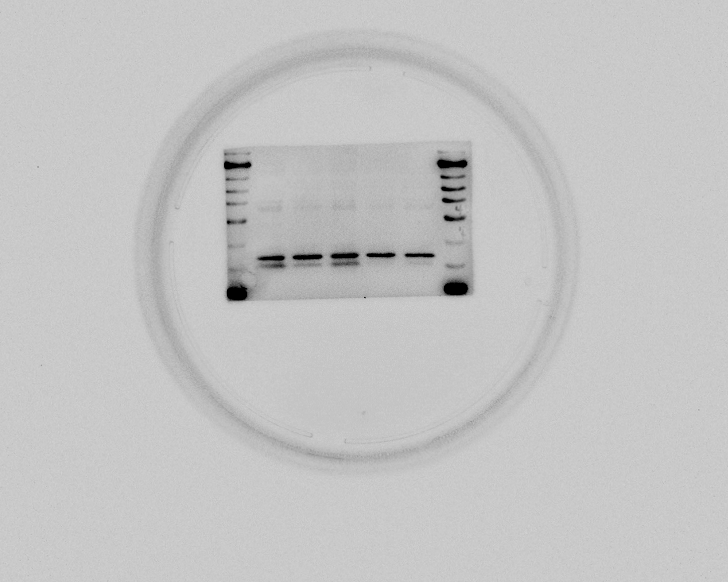

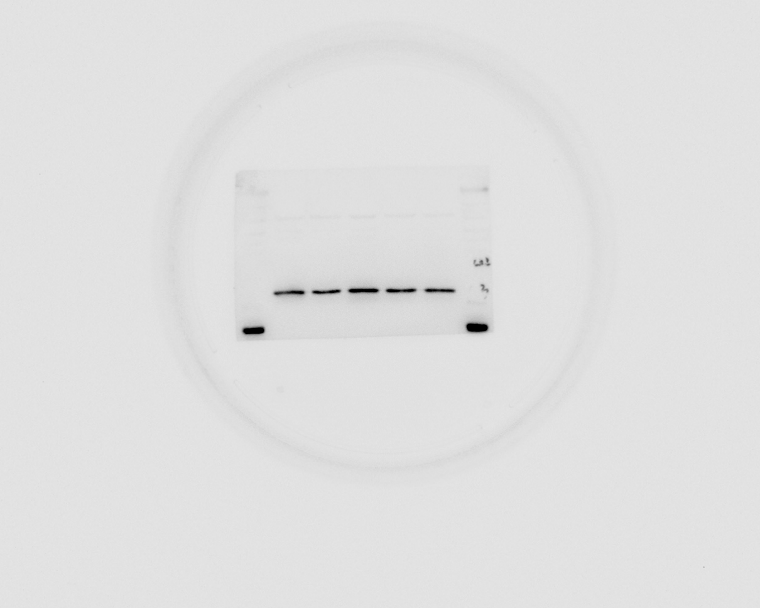


GAPDH 34KD


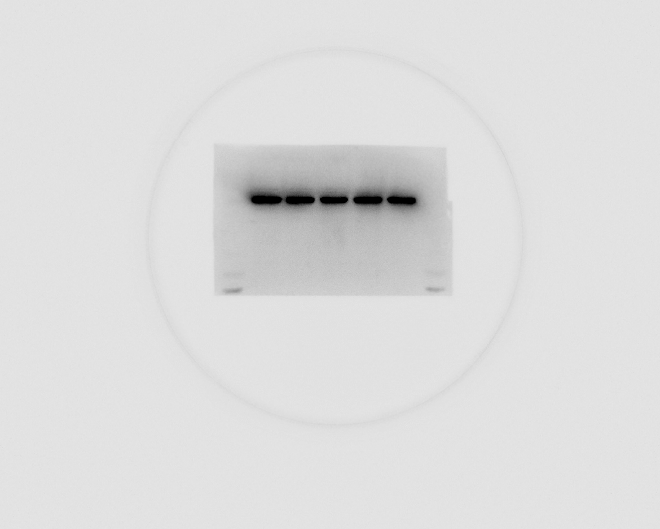

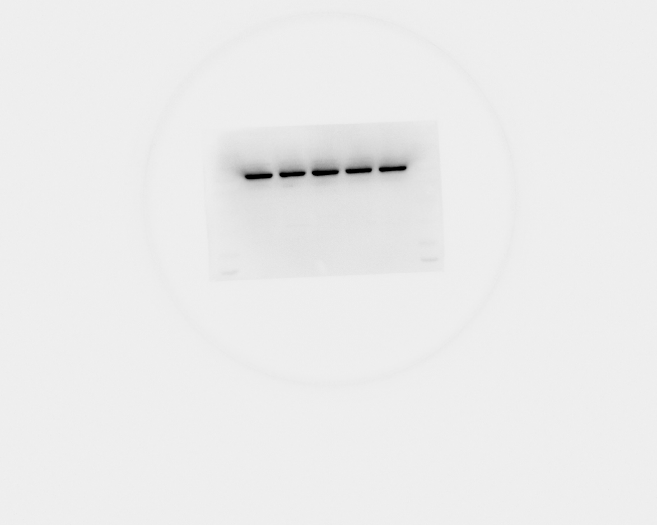

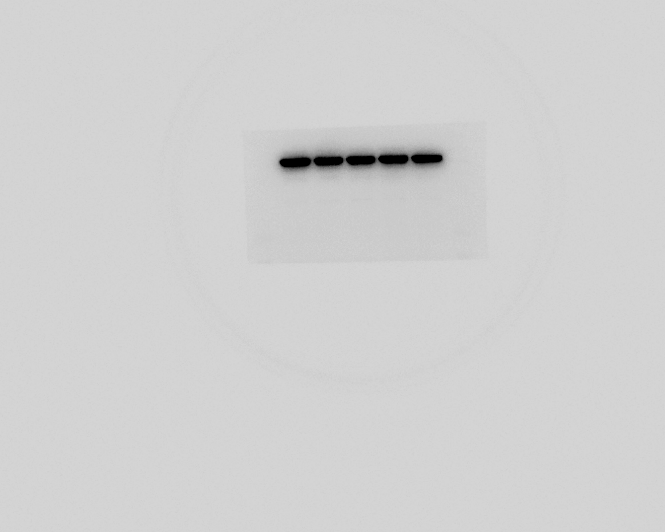


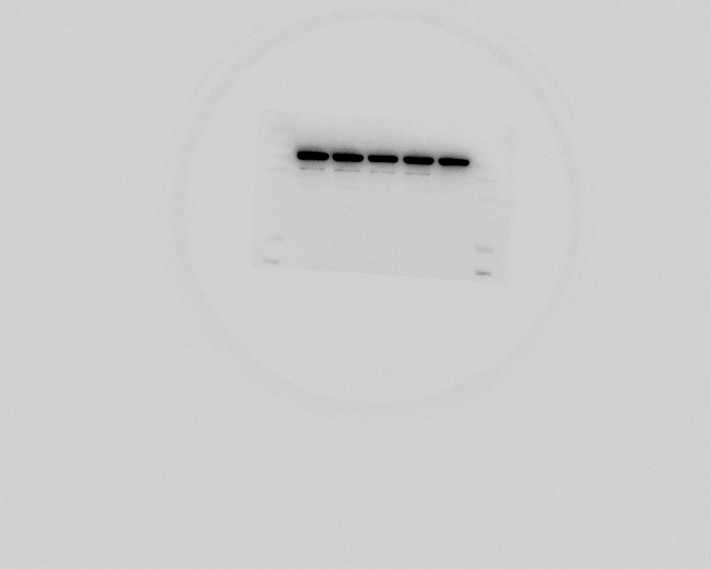

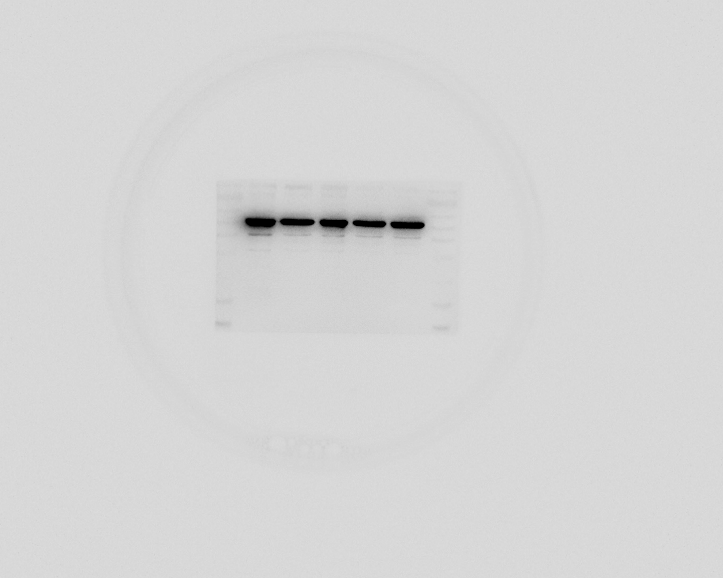

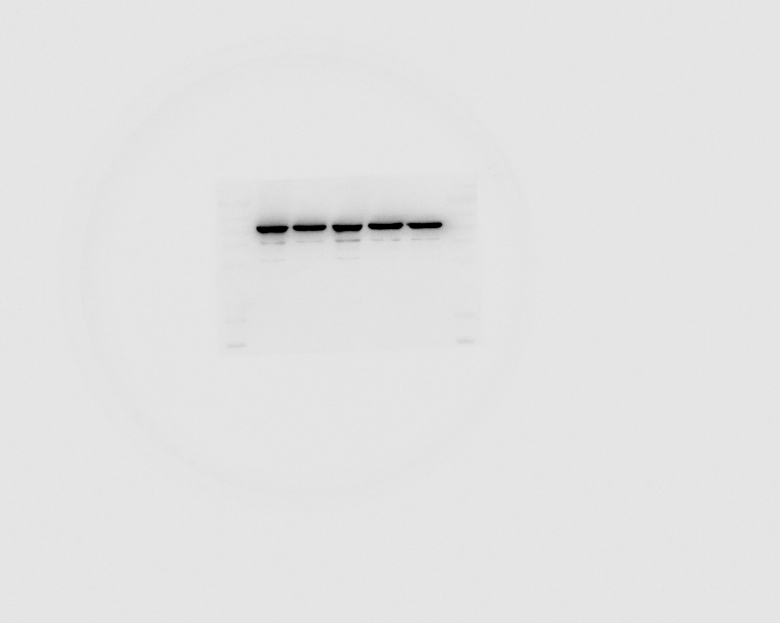


Fig.4A n=6


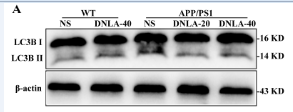


LC3 I 16 KD and LC3 II 14KD


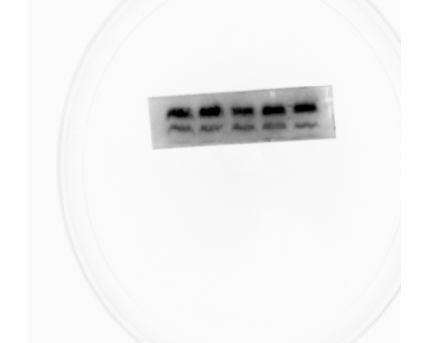

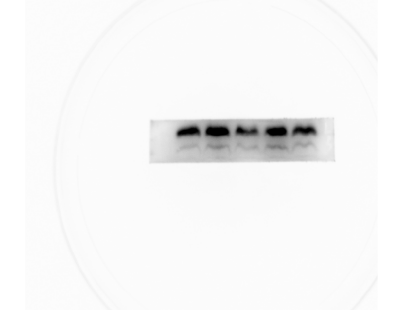

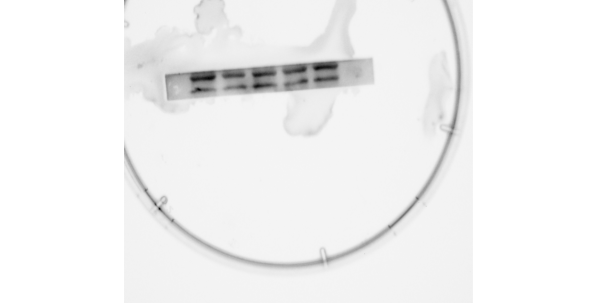


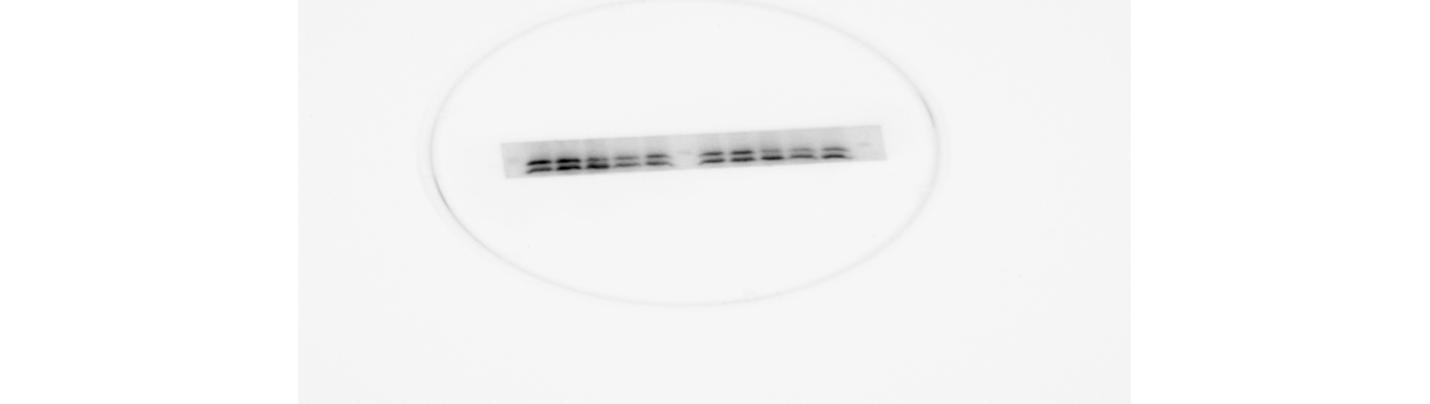

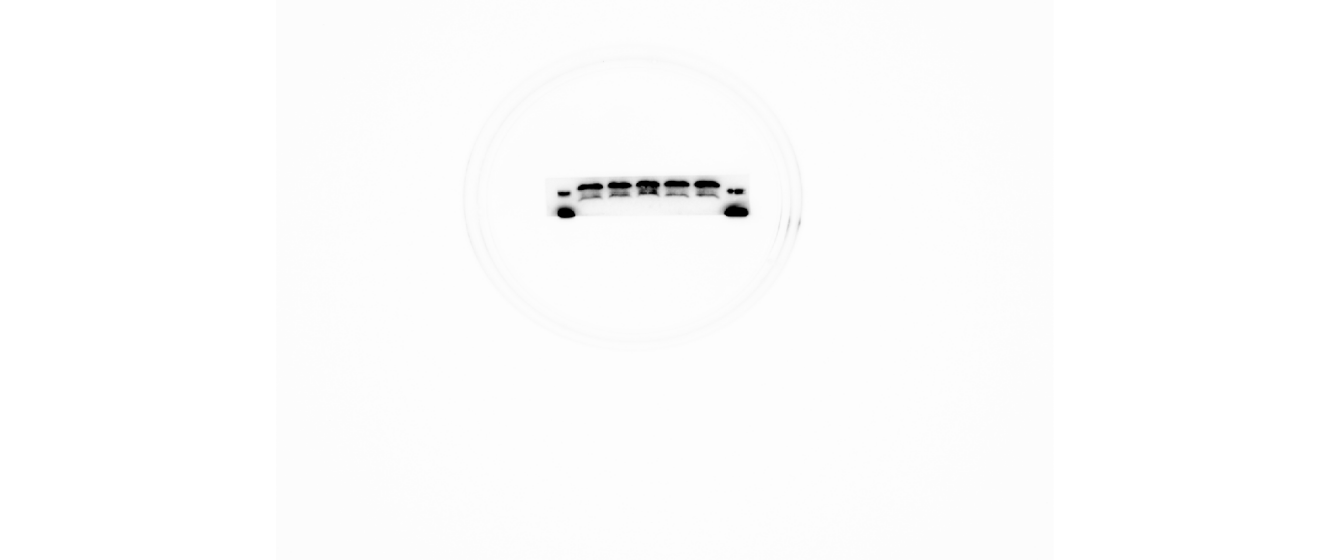


β-actin 43KD


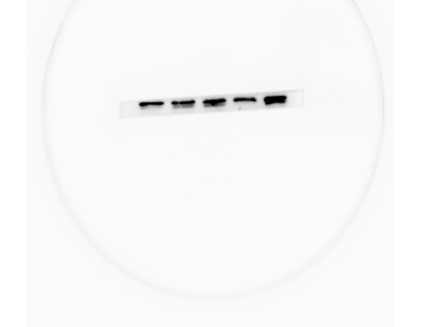

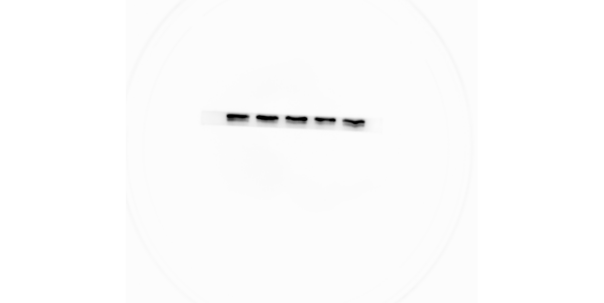

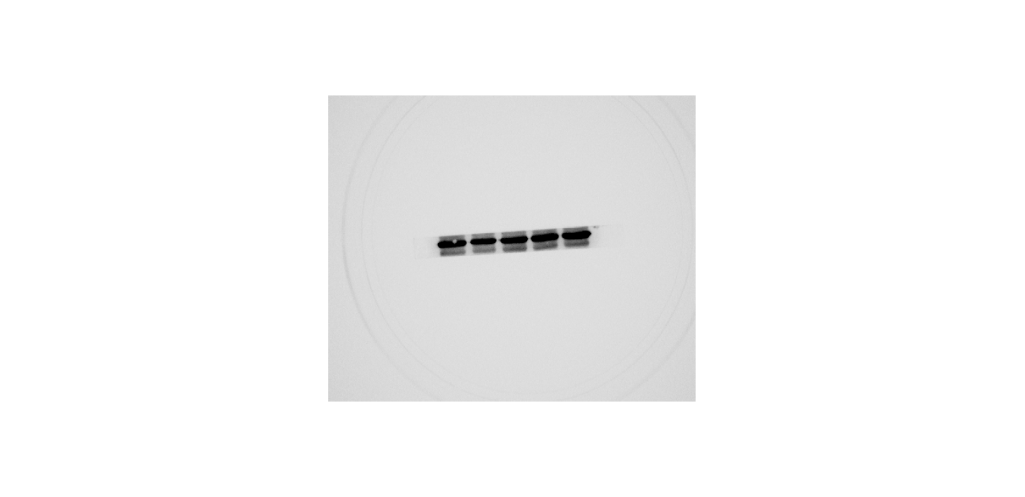


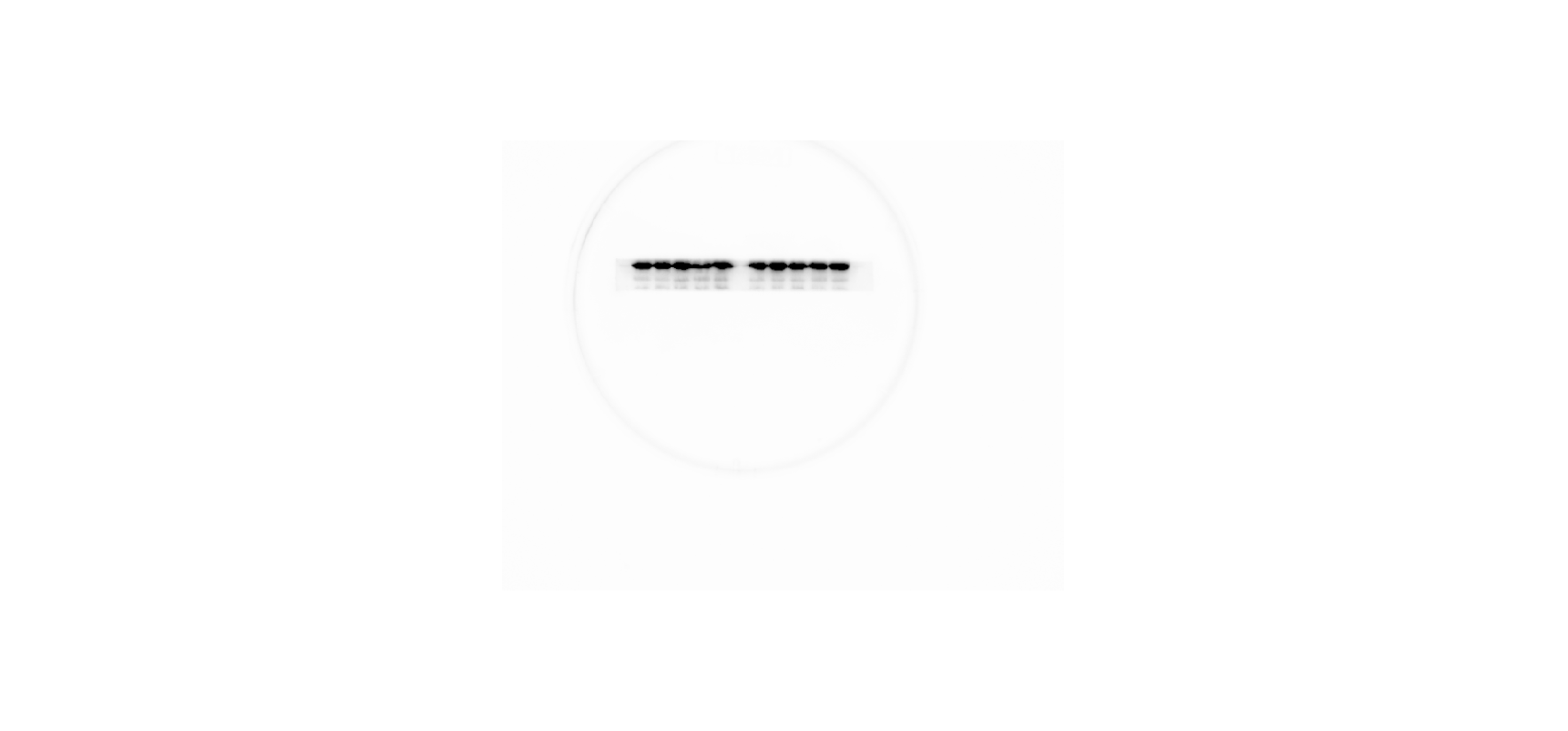

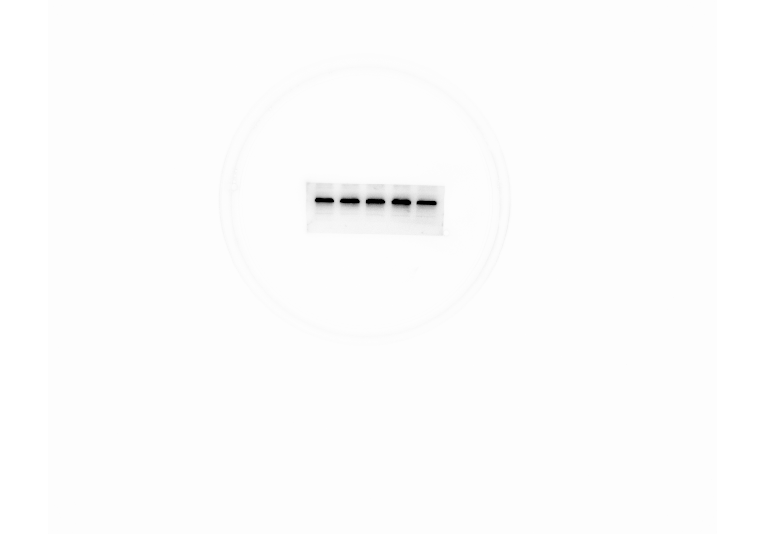


Fig.4B n=6


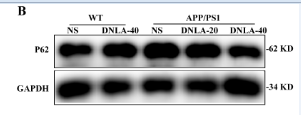


P62 62KD


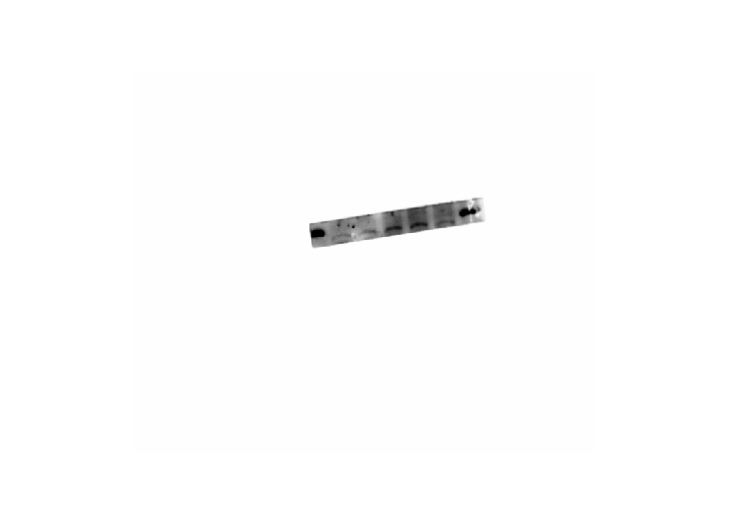

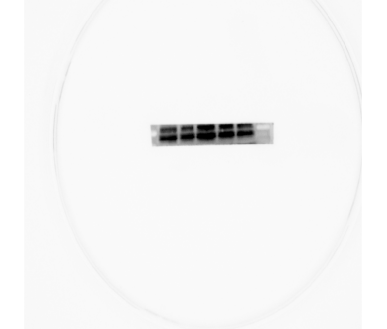

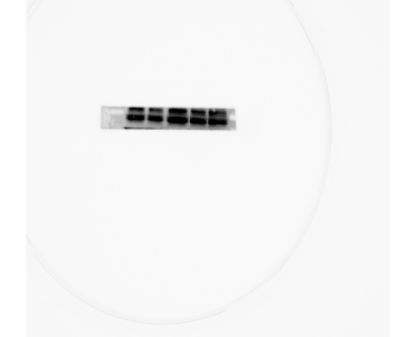

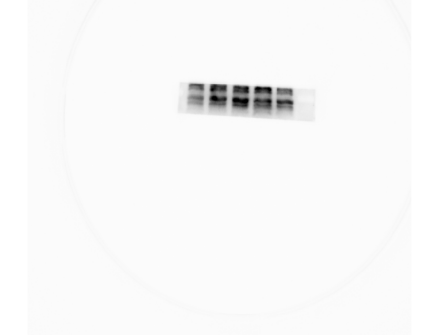


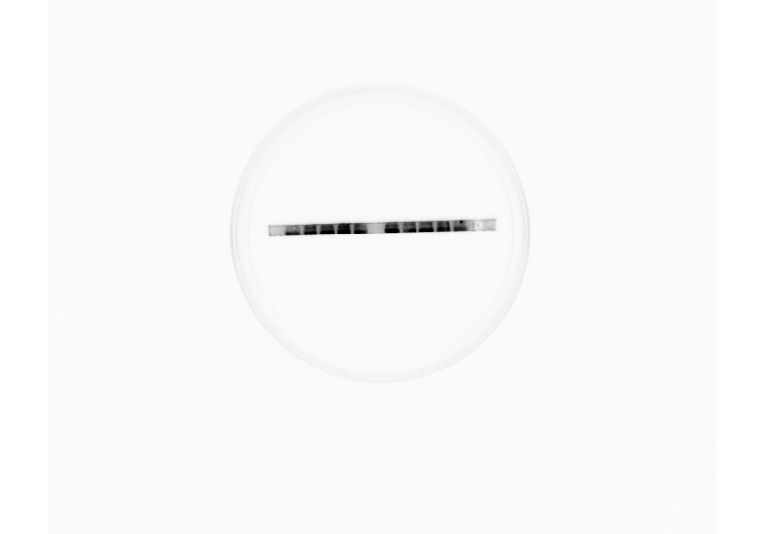

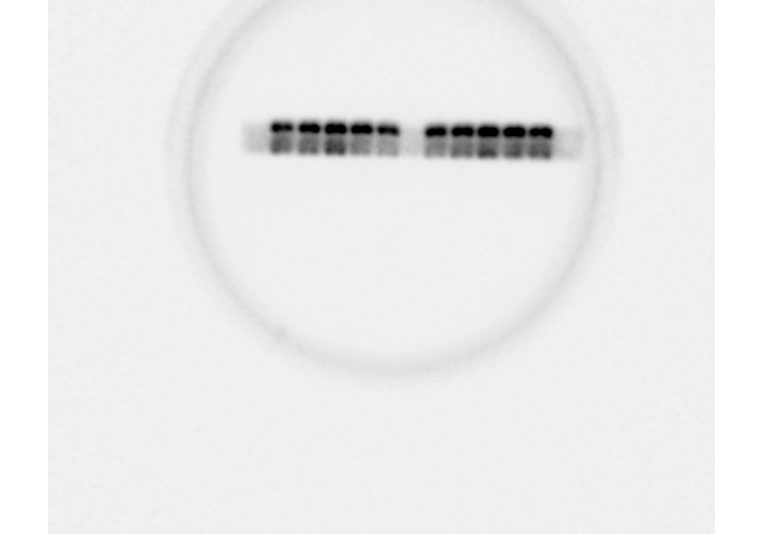


GAPDH 34KD


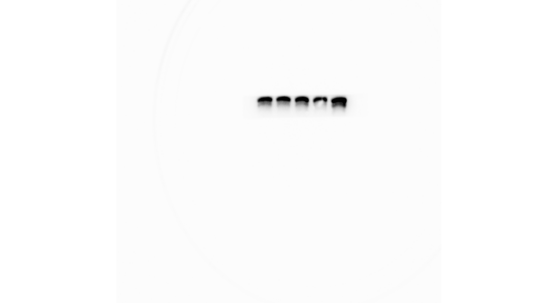

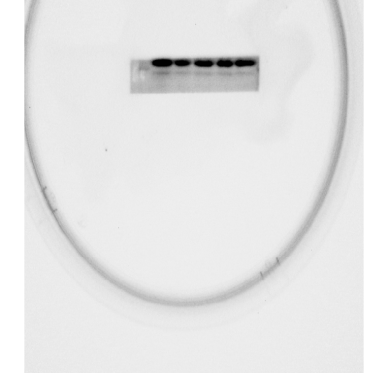

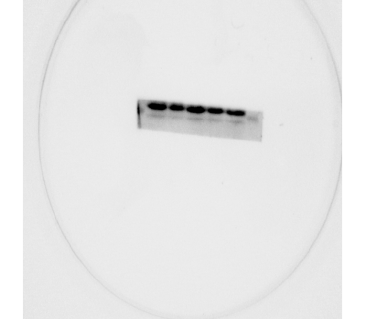

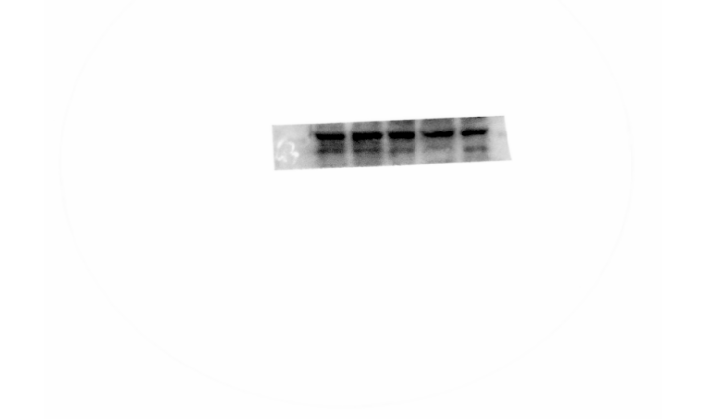


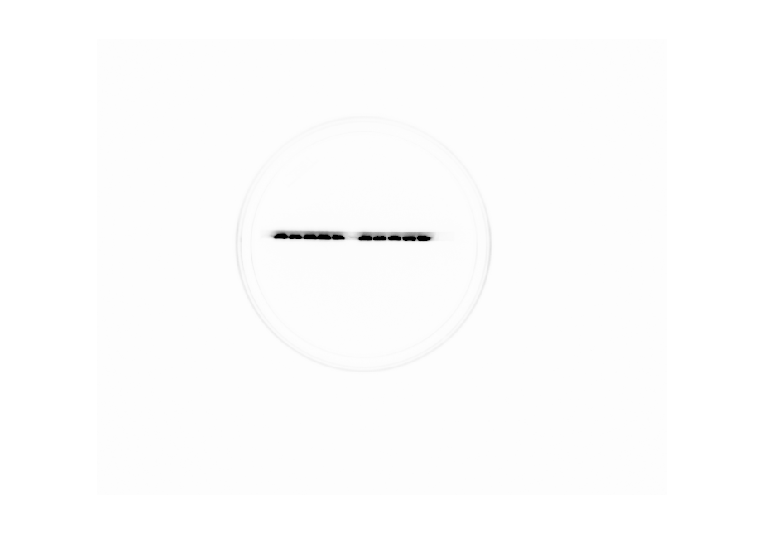

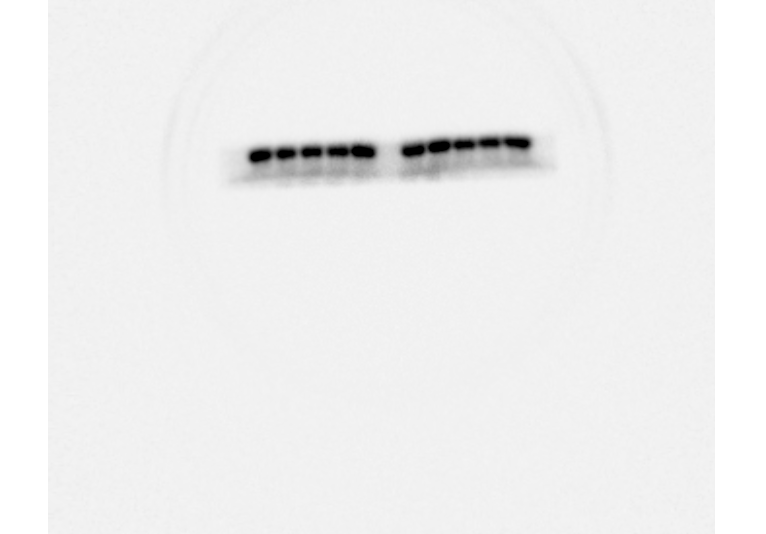


Fig.4F n=6


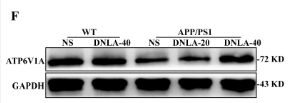


ATP6V1A 72KD


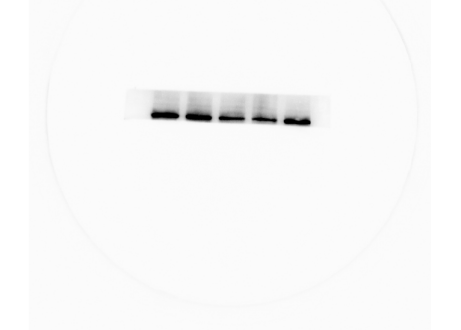

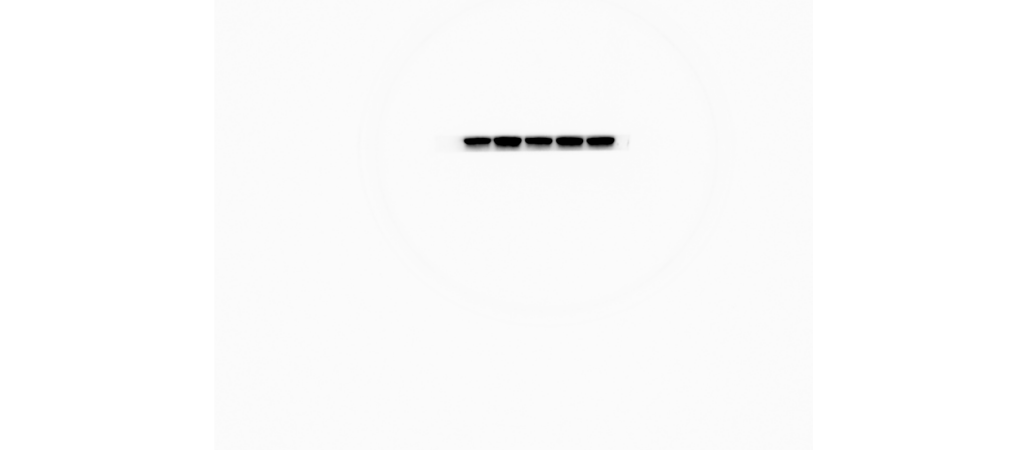

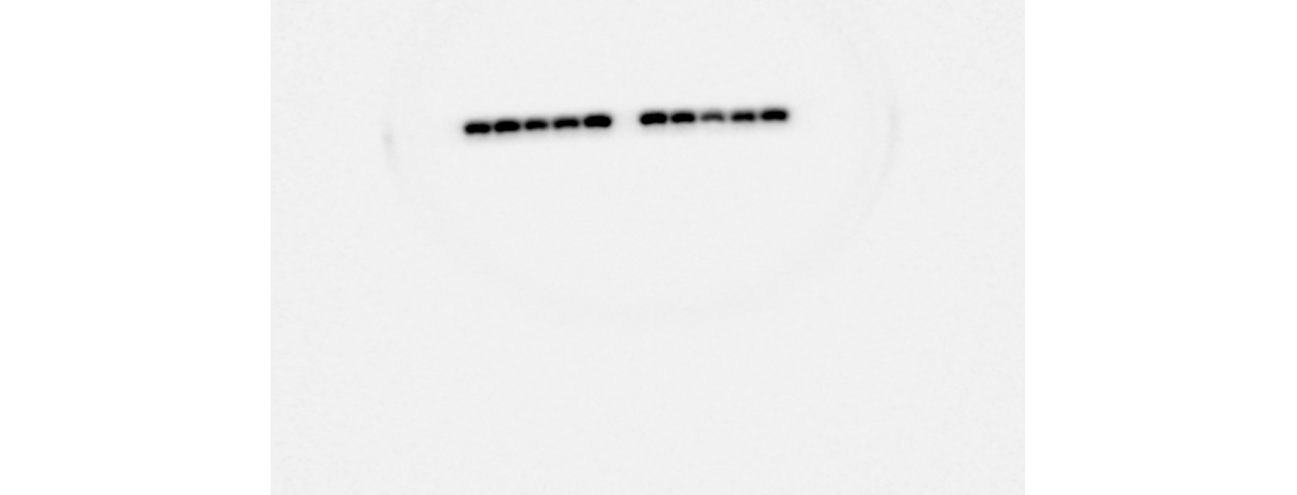


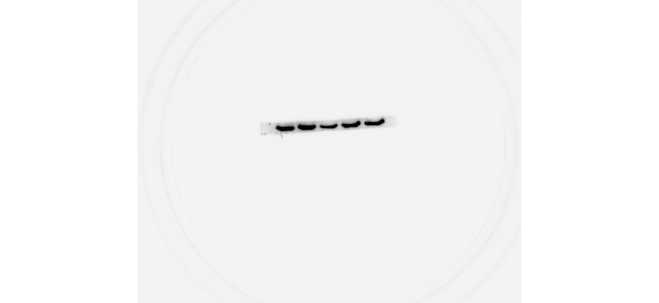

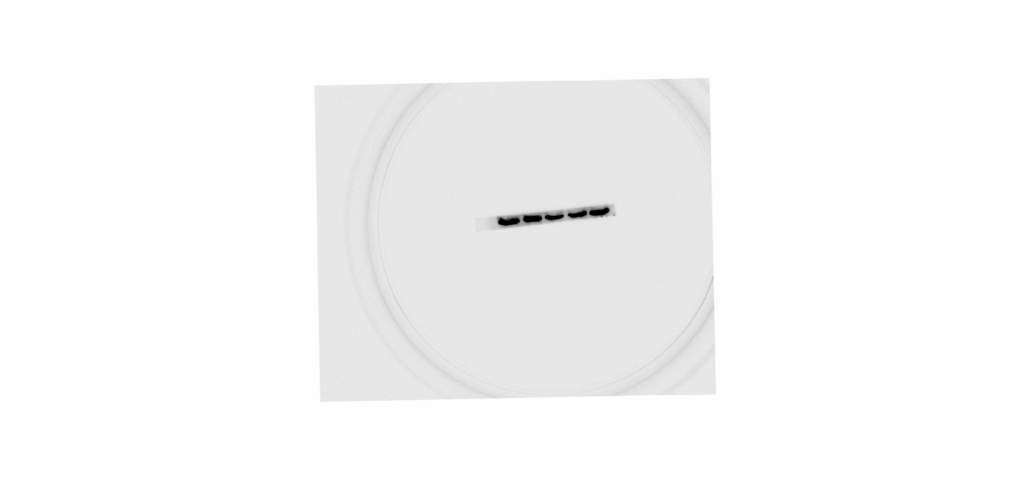

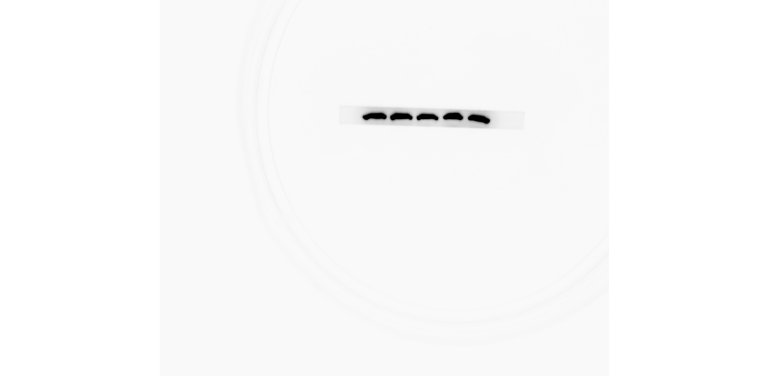


GAPDH 34KD


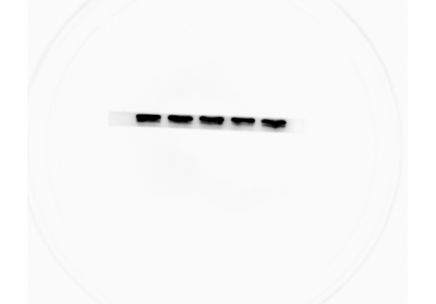

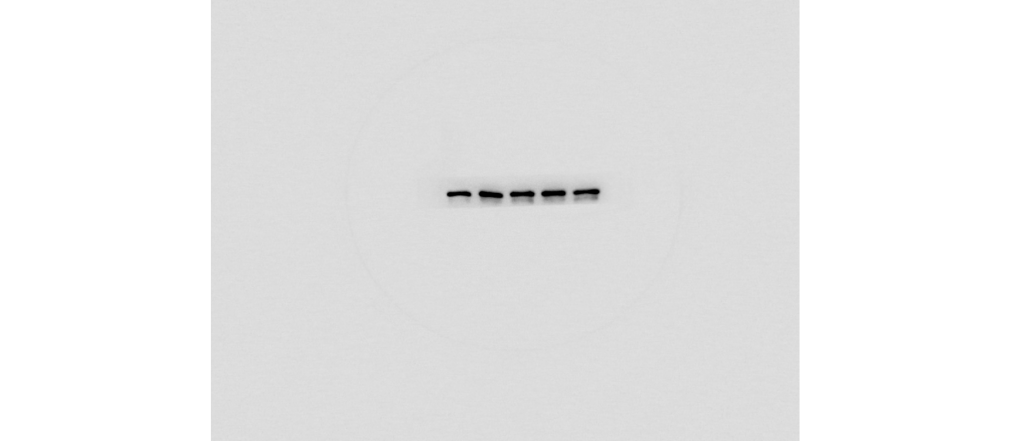

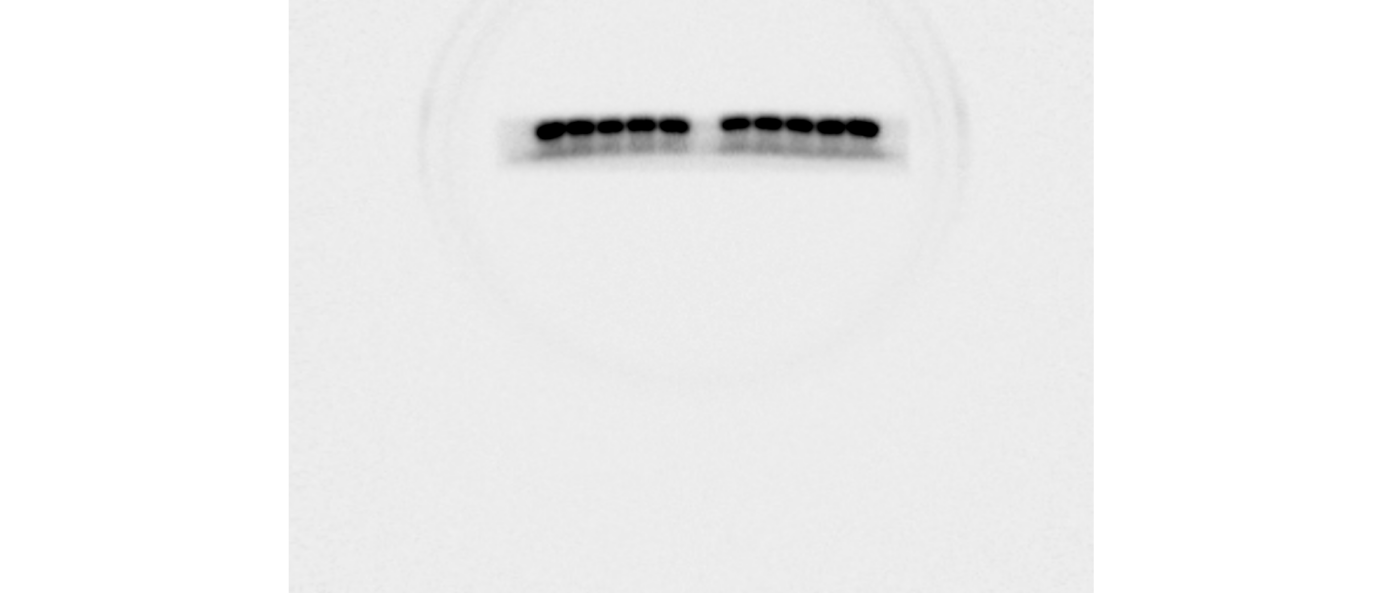


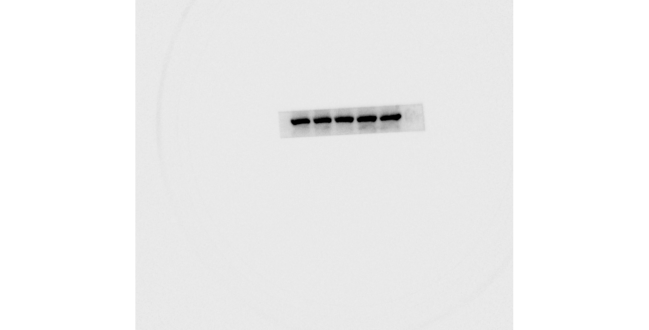

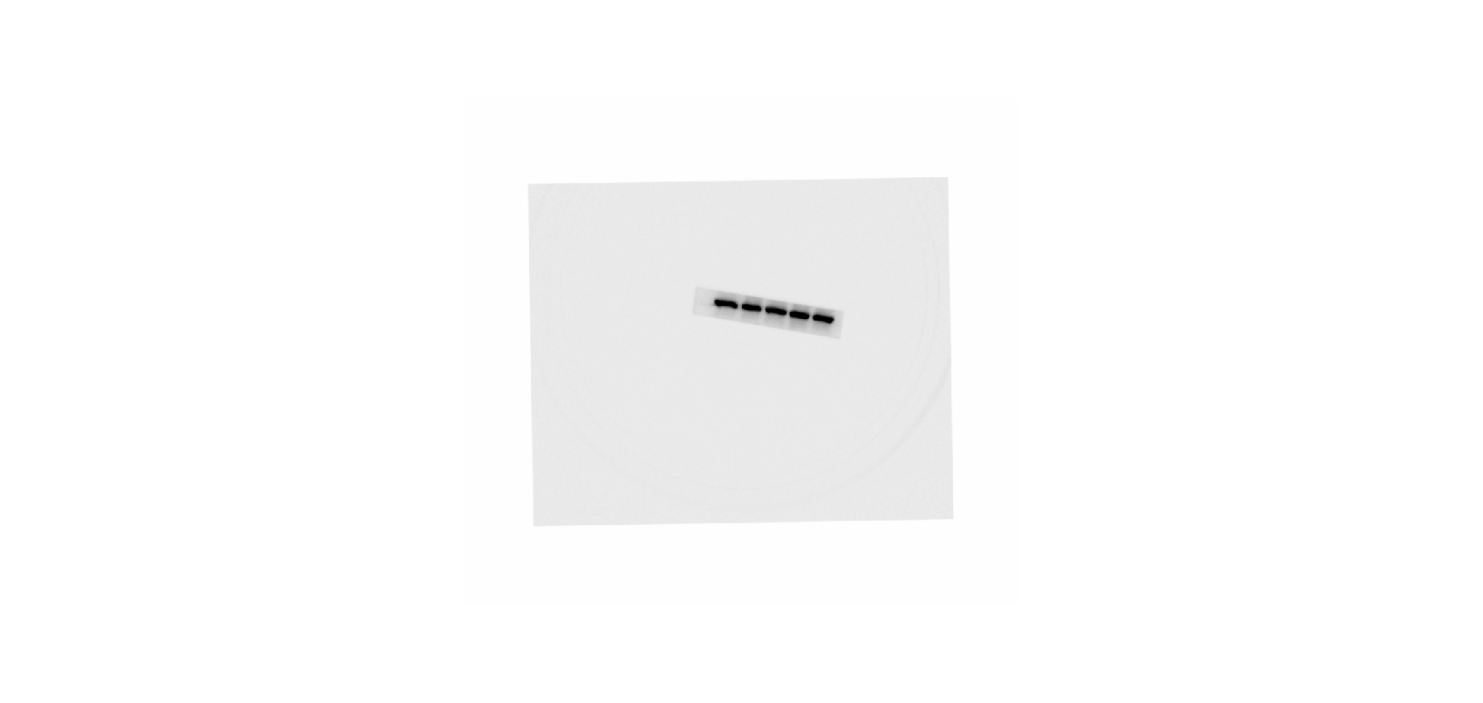

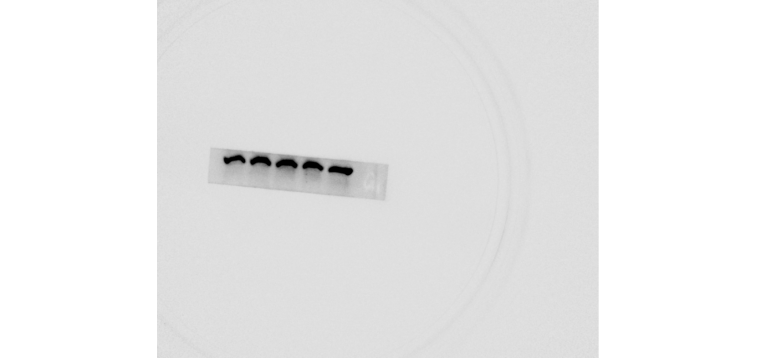


Fig.4F n=6


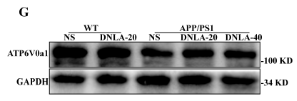


ATP6V0A1 120KD


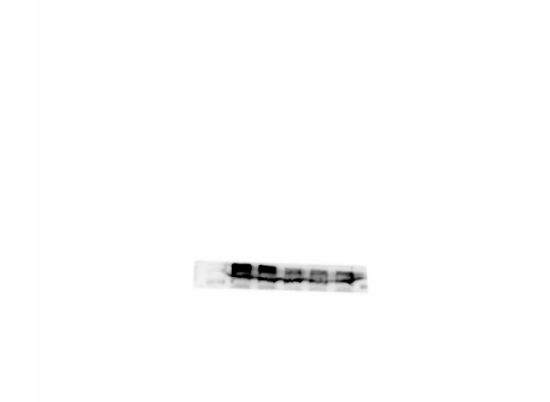

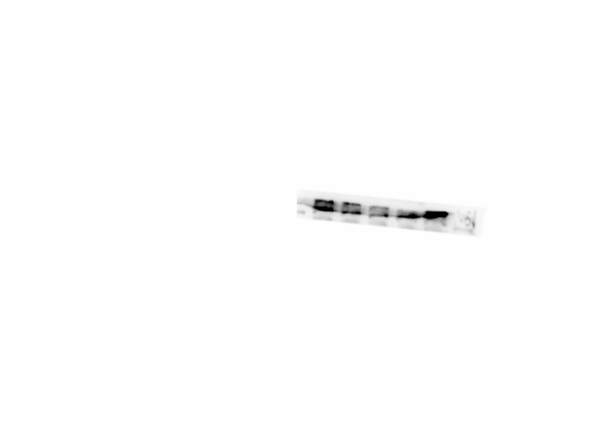

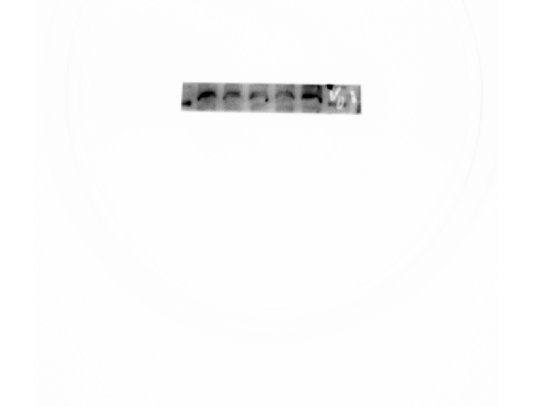


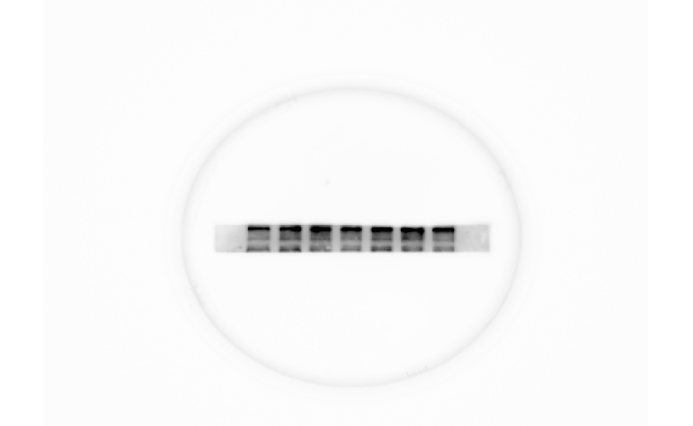


The two holes on the left of this figure are hippocampus samples of 4-month-old WT and APP/PS1 mice, which were taken for comparison with 9-month-old mice


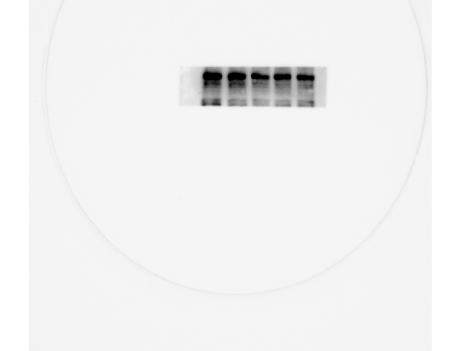

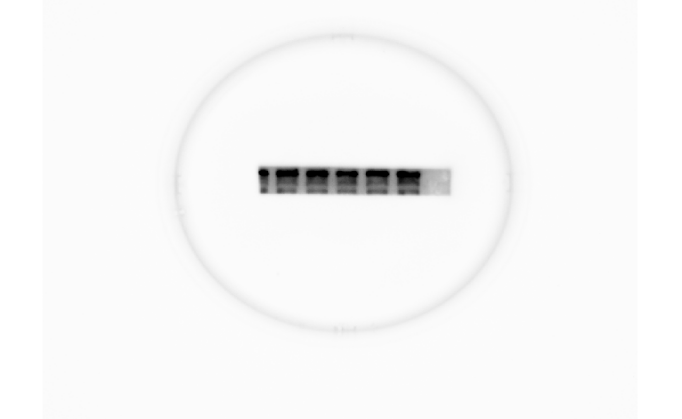


GAPDH 34KD


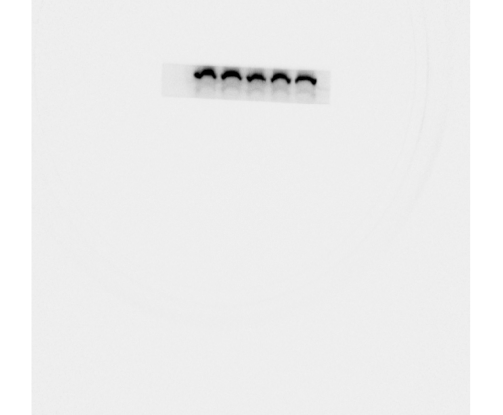

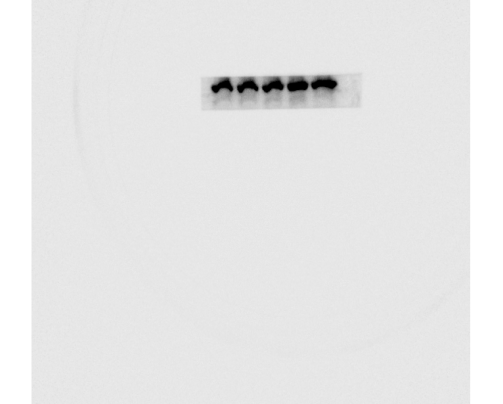

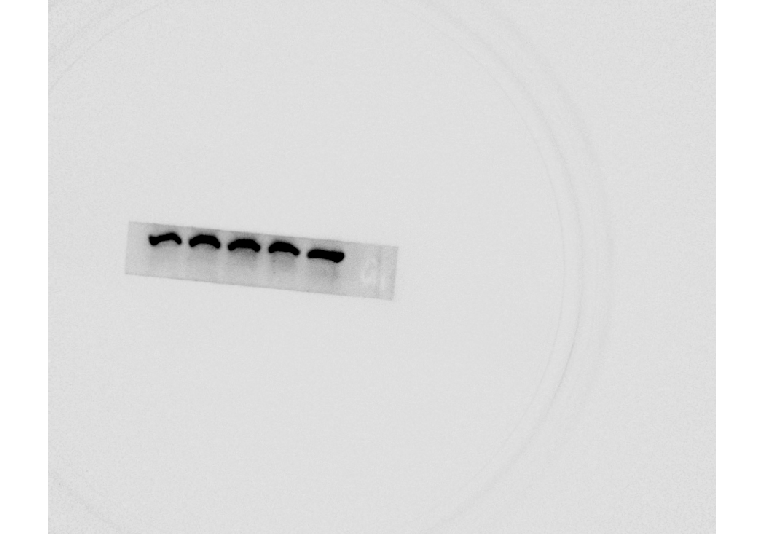


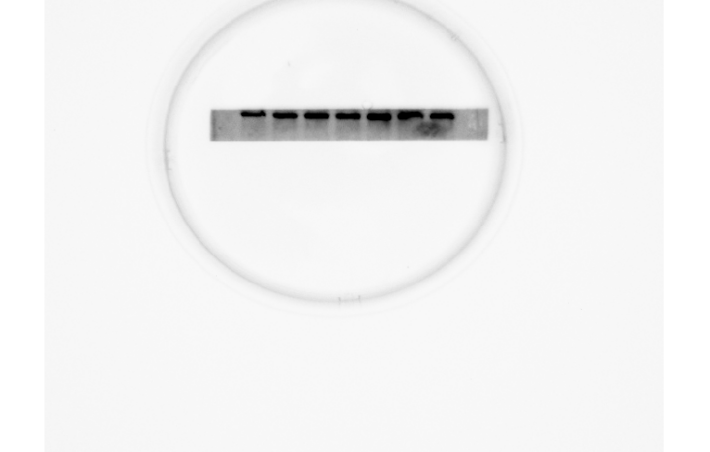

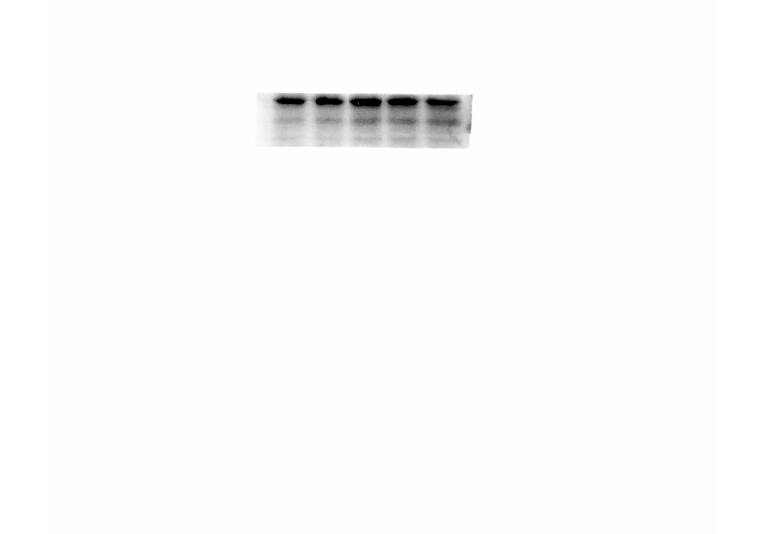

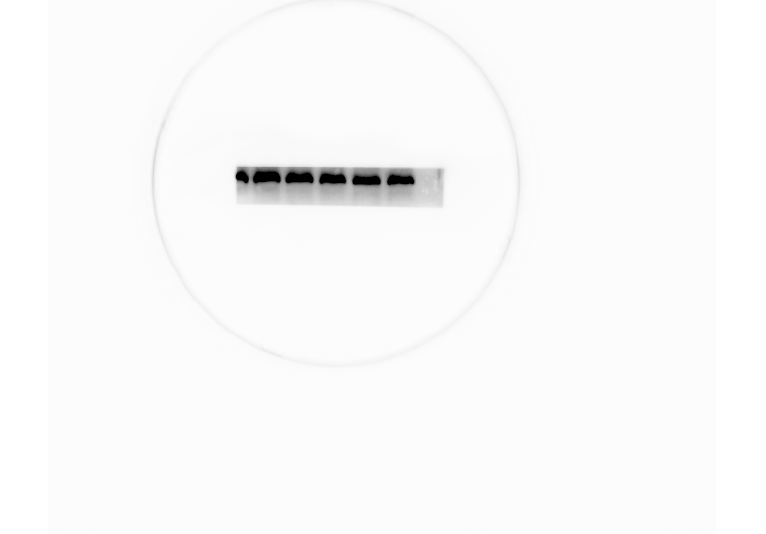


The two holes on the left of this figure are hippocampus samples of 4-month-old WT and APP/PS1 mice, which were taken for comparison with 9-month-old mice

Fig.5D n=4


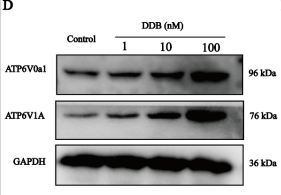


ATP6V0a1 96KD

ATP6V1A 76KD

GAPDH 36KD

Fig.6B n=3

TFEB（Cytoplasmic） 53KD

GAPDH 36KD

Fig.6C n=3

TFEB（Nucler） 53KD

PCNA 34KD

Fig.7B n=5

p-mTOR 289KD

mTOR 289KD

Fig.7C n=5

p-S6K 70KD

S6K 72KD

Fig.7D n=5

FKBP12 12KD

GAPDH 36KD

Fig.7E n=5

mTOR 289KD

GAPDH 36KD

Fig.8B n=5

ATP6V1A 68KD

GAPDH 36KD

Fig.9A n=6

TFEB(nuclear) 62KD

PCNA 34KD

Fig.9B n=6

TFEB(total) 62KD

β-actin 43KD

Fig.9F n=3

ATP6V1A 72KD

β-actin 43KD

Fig.S2 The loading sequence is Contol、DNLA 、DDB、 Contol, and I only calculated the left 3 columns.

ATP6V1A 72KD

β-actin 43KD
